# Molecular subtyping based on TRP family and prognostic assessment for TRP-associated lncRNAs in pancreatic adenocarcinoma

**DOI:** 10.1186/s12876-022-02552-y

**Published:** 2022-11-12

**Authors:** Lincheng Li, Zhaohui Xiao, Pengyi He, Wenbo Zou, Zhaoda Deng, Gong Zhang, Rong Liu

**Affiliations:** 1 Medical School of Chinese PLA, Beijing, China; 2 Faculty of Hepato-Pancreato-Biliary Surgery, the First Medical Center, Chinese PLA General Hospital, Beijing, China; 3 Translocational Medicine Research Center, Medical Innovation Research Division of the Chinese PLA General Hospital, Beijing, China; 4 Department of General Surgery, No.924 Hospital of PLA Joint Logistic Support Force, Guilin, China

**Keywords:** TRP family, lncRNA, Immune microenvironment, Pancreatic adenocarcinoma

## Abstract

**Background:**

Transient receptor potential (TRP) channels have high permeability to Ca2^+^ ions because they are non-selective ion channels. TRP channels have been implicated in tumor onset and progression, proliferation, and migration in recent years. However, the prognostic value of genes related to TRP and their specific mechanism in pancreatic adenocarcinoma (PAAD) are yet to be understood.

**Methods:**

Public databases such as TCGA and GEO were used to retrieve data on gene expression and clinical information of patients with pancreatic adenocarcinoma for our study. ConsensusClusterPlus package was used for unsupervised clustering analysis. The microenvironment cell population (MCP)-counter approach was employed to measure the immune cells infiltration status. The Pearson correlation was performed to identify TRP-associated lncRNAs.

**Results:**

Initially, we separated PAAD patients into three clusters depending on TRP-related genes, and of the three clusters, cluster B showed the least immune cell infiltration, which was correlated with poor prognosis. Moreover, GSVA enrichment analysis further revealed that cluster A was subjected to a considerable enrichment in carcinogenic signaling pathways, whereas cluster C was enriched in immune-related pathways. Then, using TRP-associated lncRNAs as a starting point, we constructed a prognostic risk model for PAAD patients that could efficiently predict their prognosis. Further, GSEA revealed that cancer-related pathways, for instance, the cell cycle, p53 signaling pathway, etc. were considerably enriched in the high-risk group. In addition, we looked into the link between the prognostic model and the immunological microenvironment. Lower cytotoxic lymphocytes, NK cells, CD8 T cells, and endothelial cells infiltration were found to be associated with high risk using the MCP-counter algorithm. The expression of CD274, POLE2, MCM6, and LOXL2 was also found to be higher in the high-risk group. TMB was also considerably greater in high-risk individuals, indicating that immune checkpoint inhibitors (ICIs) therapy may benefit them more. Lastly, qRT-PCR further confirmed the differential expression of these prognostic TRP-associated lncRNAs, indicating that these lncRNAs play an imperative role in PAAD tumorigenesis.

**Conclusion:**

TRP family genes may represent a new class of candidate molecular markers of the occurrence and progression of PAAD. Risk models based on TRP-associated lncRNAs could provide important new references for immunotargeted therapy of pancreatic adenocarcinoma.

**Supplementary Information:**

The online version contains supplementary material available at 10.1186/s12876-022-02552-y.

## Introduction

The onset of pancreatic adenocarcinoma (PAAD) is insidious with no prominent symptoms at the early stage. 80% of the patients with PAAD have reached a locally advanced stage or showed distant metastasis at the time of diagnosis, leading to the loss of a chance to undergo radical surgery [1]. Pancreatic adenocarcinoma has a high mortality rate due to its extreme aggressiveness and a five-year survival rate of < 10% [2]. The currently used chemoradiotherapy method to treat PAAD patients with locally advanced or distant metastasis does not significantly improve prognosis [3]. To enhance the prognosis, it is crucial to develop an effective treatment. Targeted therapy, as a new therapeutic approach, is critical in the treatment of pancreatic adenocarcinoma.

TRP channels, also known as transient receptor potential channels, make up a multifunctional family of ion channels that are recently discovered and mostly calcium-permeable [4]. Research has revealed that the expression of various TRP proteins in numerous cancer types has altered and TRP ion channels play a crucial role in tumor formation, proliferation, and migration [5–7]. TRP channel family members have also been reported as a good prognostic marker and a target for cancer drug therapy in recent decades [8]. Recently, Pagano et al. [9] showed that TRPM8 is significantly upregulated in CRC, and promotes tumor growth by activating Wnt/β-catenin signaling pathway. And in hepatocellular carcinoma, HC-067047, a TRPV4 antagonist, could suppressed cell proliferation, induced apoptosis and decreased the migration capability by attenuating the EMT process [10]. TRPV1, TRPV4, TRPV6, TRPC1, TRPC3, TRPC6, TRPM2, TRPM7, TRPM8, TRPML1, and TRPML are TRP-related genes that have been linked to pancreatic adenocarcinoma [11]. Studies have shown that high TRPM8 expression was significantly correlated with large tumor size, advanced TNM and distant metastasis in pancreatic adenocarcinoma [12]. And a recent bioinformatic analysis showed that extensive genetic alterations in TRPV channel-related genes may play an important role in cancer progression, including PAAD [13]. In addition, TRPA1 has been shown to be involved in pancreatic cancer cell migration as well as cell cycle progression through non-selective calcium trafficking [14]. Thus, TRP family could be a target for cancer therapy, allowing for more effective treatment strategies for cancer patients and better patient outcomes.

Based on TRP family genes, we categorized PAAD patients into three subtypes. Then, using TRP-associated lncRNAs, we constructed a PAAD-related prognostic risk model that had the potential to predict the prognosis of PAAD patients in an accurate manner. We also investigated immune infiltration and immune checkpoint expression and found that the high-risk group may benefit more from ICIs therapy.

## Materials and methods

### Pancreatic adenocarcinoma datasets source and preprocessing

The Cancer Genome Atlas (TCGA) and Gene Expression Omnibus (GEO) databases were used to acquire public transcriptome and clinical data. In this investigation, we obtained TCGA-PAAD (177 patients) and GSE62452 (69 patients) for further analysis. RNA sequencing data (FPKM value) was retrieved for TCGA-PAAD and then converted into TPM values. We directly obtained the normalized matrix files for the GSE62452 cohort. For batch correction of the combined matrix, the sva package’s “ComBat” algorithm was employed. Data on somatic mutation was obtained from the TCGA database.

### Unsupervised clustering for 28 TRP-related genes

Using the ConsensusClusterPlus package, unsupervised clustering analysis was used to categorize various subtypes on the basis of the expression levels of 28 TRP-related genes. The partition around medoids (PAM) algorithm with Euclidean distance was employed for cluster analysis and 80% of the samples were resampled 50 times. The empirical cumulative distribution function plot was employed for finding the optimal number of clusters.

### Gene set variation analysis (GSVA) enrichment analysis

We used “GSVA” R packages to carry out GSVA enrichment analysis to investigate the biological processes implicated in distinct clusters. The “c2.cp.kegg.v7.2.symbols” gene sets used for the enrichment study were provided by the Molecular Signatures Database-MsigDB. A *P* value < 0.05 indicated a statistically significant difference.

### Immune cell infiltration analysis

The relative abundance of immune cell infiltration in PAAD was quantified with a single-sample gene-set enrichment analysis (ssGSEA) algorithm. Moreover, the microenvironment cell population (MCP)-counter approach was also employed to measure the immune cells’ infiltration status, which was examined with RNA sequencing data to determine tumor tissue immunity and stromal cell invasion [15].

### Identification of TRP-associated lncRNAs

We used mRNA expression data to retrieve the lncRNA matrix. Subsequently, the Pearson correlation identified the link between the lncRNAs and TRP-related genes. The lncRNAs with correlation coefficient |Rho| > 0.4 and *p <* 0.001 were defined as TRP-associated lncRNAs (TRlncRNAs).

### Construction and validation of a TRP-associated lncRNAs prognostic signature

The “survival” R package helped in a Univariate Cox regression analysis to identify prognostic TRP-associated lncRNAs. To authenticate this study further, the “caret” R package was used to randomly categorize TCGA-PAAD into 2 sets; a training set consisting of 108 samples and an internal validation set comprising 69 samples in a ratio of 3:2. Table 1 displays the clinical traits. Afterward, multivariate Cox regression analysis calculated the risk coefficients of the individual prognostic gene. The equation for risk score is as follows: Risk score = ΣCoef TRlncRNAs × Exp TRlncRNAs. The Coef TRlncRNAs demonstrate the coefficient of each TRlncRNAs and Exp TRlncRNAs show the expression of each TRlncRNAs. The samples were categorized into high and low-risk groups by the median risk score, and we drew the survival curve using the “survival” “survminer” R package. With the help of AUC, an accuracy comparison of prognostic models was performed using the “survivalROC” R package.

**Table 1:** Characteristics of the datasets

Variable	Total	Train	Test	P-value
Age				
< =65	93 (52.54%)	57 (52.78%)	36 (52.17%)	1
> 65	84 (47.46%)	51 (47.22%)	33 (47.83%)	
Gender				
FEMALE	80 (45.2%)	47 (43.52%)	33 (47.83%)	0.6842
MALE	97 (54.8%)	61 (56.48%)	36 (52.17%)	
Grade				
G1	31 (17.51%)	15 (13.89%)	16 (23.19%)	0.42
G2	94 (53.11%)	61 (56.48%)	33 (47.83%)	
G3	48 (27.12%)	30 (27.78%)	18 (26.09%)	
G4	2 (1.13%)	1 (0.93%)	1 (1.45%)	
unknow	2 (1.13%)	1 (0.93%)	1 (1.45%)	
Stage				
Stage I	21 (11.86%)	14 (12.96%)	7 (10.14%)	0.6667
Stage II	146 (82.49%)	89 (82.41%)	57 (82.61%)	
Stage III	3 (1.69%)	1 (0.93%)	2 (2.9%)	
Stage IV	4 (2.26%)	3 (2.78%)	1 (1.45%)	
unknow	3 (1.69%)	1 (0.93%)	2 (2.9%)	
T				
T1	7 (3.95%)	6 (5.56%)	1 (1.45%)	0.3764
T2	24 (13.56%)	16 (14.81%)	8 (11.59%)	
T3	141 (79.66%)	85 (78.7%)	56 (81.16%)	
T4	3 (1.69%)	1 (0.93%)	2 (2.9%)	
unknow	2 (1.13%)	0 (0%)	2 (2.9%)	
M				
M0	79 (44.63%)	52 (48.15%)	27 (39.13%)	1
M1	4 (2.26%)	3 (2.78%)	1 (1.45%)	
unknow	94 (53.11%)	53 (49.07%)	41 (59.42%)	
N				
N0	49 (27.68%)	34 (31.48%)	15 (21.74%)	0.214
N1	123 (69.49%)	71 (65.74%)	52 (75.36%)	
unknow	5 (2.82%)	3 (2.78%)	2 (2.9%)	

### The nomogram establishing

We drew a nomogram using the “rms” R package on the basis of clinical factors. For evaluating the consistency of the actual and predicted survival, we further drew calibration curves for a reliability check.

### Validation of prognostic TRP-associated lncRNAs using qRT-PCR

HPDE6-C7, BXPC-3, SW1990 and PANC-1 cell lines were purchased from the American Type Culture Collection (ATCC, Manassas, VA, USA). We then cultured cells in either RPMI 1640 or DMEM supplemented with 10% fetal bovine serum (FBS, Gibco), and placed them in a 37 °C, 5% CO2 incubator. The PAAD tumors and normal tissues were collected from inpatients in hepatobiliary Surgery department of Chinese PLA general Hospital. The selected cases were diagnosed as pancreatic adenocarcinoma by two pathologists. Perform qRT-PCR to detect gene expression changes in prognostic TRP-associated lncRNAs. Specifically, we isolated total RNA from cancer and normal samples utilizing TRIzol reagent (Ambion) and then converted it into cDNA using Eppendorf Mastercycler®. StepOnePlus Real-Time PCR System was utilized to execute qPCR premised on the primers listed in Table 2.

**Table 2:** Primers used for qRT-PCR analysis

Gene	Direction	Sequences (5′–3′)
18 s	Forward	AACCCGTTGAACCCCATT
18 s	Reverse	CCATCCAATCGGTAGTAGCG
LINC01133	Forward	TGGTGGAGAGAATGGAGG
LINC01133	Reverse	AACCCAGTTCCTTAGAATCTTC
LINC00973	Forward	TTGAAGGCTTCCTGGTCTGAG
LINC00973	Reverse	AGGCTTACATTCCAGCTGTGT
TRPC7-AS1	Forward	GCCTCCTCCTTCCATAACG
TRPC7-AS1	Reverse	CCCACAGCCTAGACCCATT
LINC01091	Forward	TAAGAAGTACGCATTCATAAGG
LINC01091	Reverse	CCCACTTTGGAGGCTTTA

### Statistical analysis

For comparing continuous data, the independent samples t-test or Wilcoxon rank-sum test was employed, and chi-square tests were used for categorical data. To examine survival differences across groups, a Kaplan–Meier analysis with a log-rank test was performed. R was used to conduct all statistical analyses.

## Result

### The landscape of genetic variation of TRP-related genes in pancreatic adenocarcinoma

**Fig. 1:**
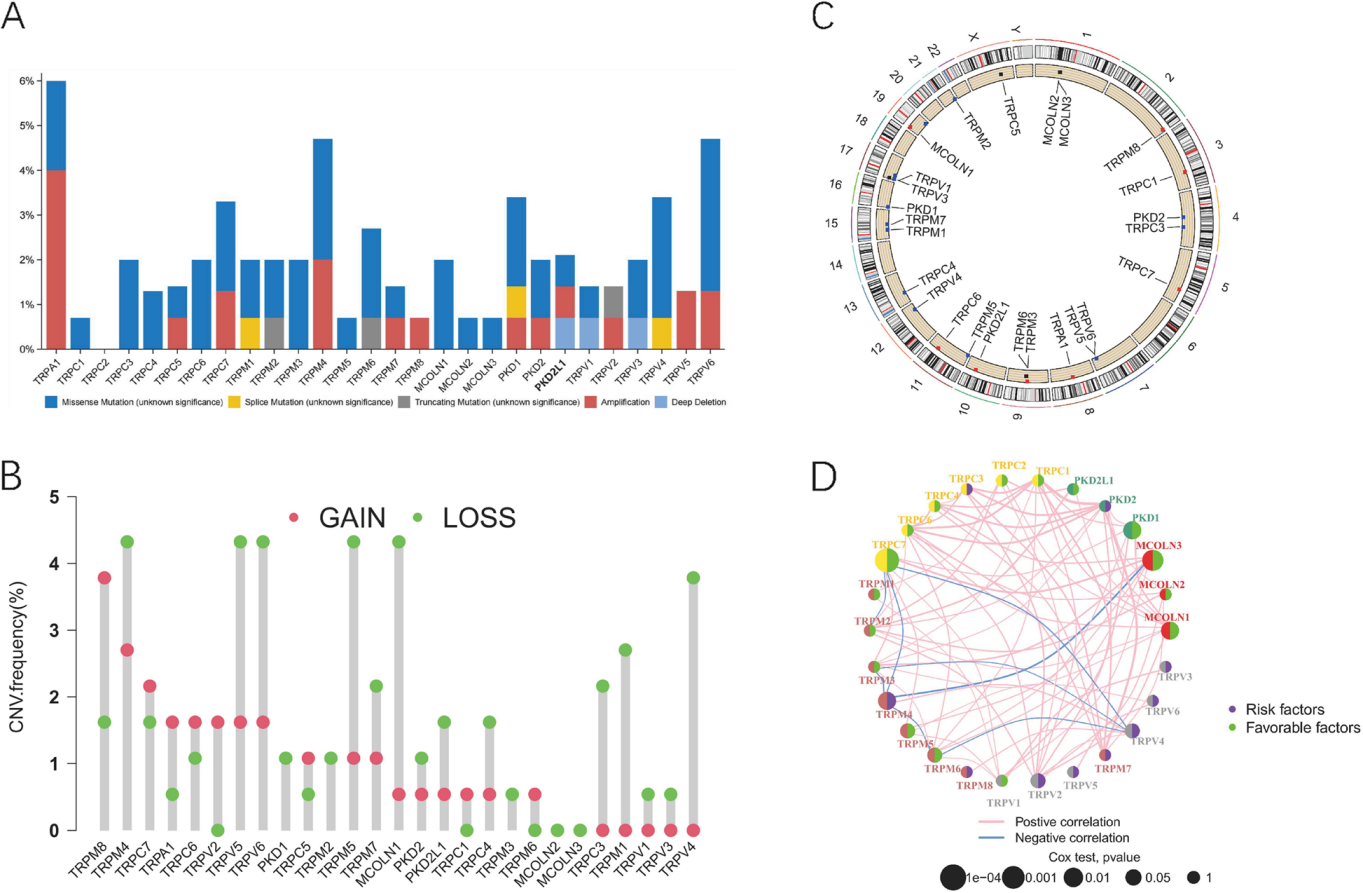
The landscape of genetic variation of TRP-related genes in pancreatic adenocarcinoma. **A**. The mutation frequency of 28 TRP family genes in TCGA-PAAD patients. **B**. The CNV variation frequency of TRP family genes in TCGA cohort. **C**. The location of CNV alteration of TRP family genes on 23 chromosomes. **D**. The interaction between 28 TRP family genes in pancreatic adenocarcinoma

At first, the incidence frequency of somatic mutations of 28 TRP family genes in PAAD was summarized based on cBioPortal database (http://cbioportal.org) [16]. TRP-related gene mutations were found in 52 of the 149 cases, with a frequency of 35%. According to the findings, the highest mutation frequency was found in TRPA1, followed by TRPM4 and TRPV6 (Fig. 1 A). The incidence of copy number variation (CNV) mutations was shown to be prevalent in 28 TRP family genes, with more number of copy number deletion genes as compared to amplification genes (Fig. 1 B). The site of CNV alteration of TRP family genes on chromosomes was illustrated in Fig. 1 C. Univariate Cox regression evaluated the prognostic significance of 28 TRP family genes in PAAD patients. A network was used to describe the interaction of TRP family genes and their effect on the prognosis of PAAD patients. We discovered that TRP family genes’ expression was significantly correlated (Fig. 1 D).

### The differential expression and prognostic value of TRP-related genes

We first explored the differential expression of TRP-related genes in pancreatic adenocarcinoma using GEPIA [17] and CCLE databases [18]. As shown in Supplementary Fig. 1, we found that TRPC1, TRPM2, TRPM4, MCOLN1, PDK2, TRPV2 and TRPV4 were highly expressed in PAAD tissues, while MCOLN3 and TRPV6 were lowly expressed. In addition, the expression of TRP-related genes in pancreatic cancer cell line was shown in Supplementary Fig. 2. More importantly, we also evaluated the prognostic value of TRP-related genes in pancreatic adenocarcinoma using the KM-plotter database (https://kmplot.com/analysis/). The result showed that TRPC1, TRPC4, TRPC5, TRPC6, TRPC7, TRPM6, MCOLN1, MCOLN3, PDK1 and TRPV1 were favorable to the prognosis of patients, while TRPV1, TRPM1, TRPM4, TRPM8 and TRPV6 were unfavorable (Supplementary Fig. 3). In addition, we constructed the PPI network based TRP channel proteins by STRING website. And we found that TRPC1 was associated with almost all TRP channel proteins, suggesting that TRPC1 may be a key molecule in TRP ion channels (Supplementary Fig. 4A-B).

### Unsupervised clustering based on 28 TRP-related genes

**Fig. 2:**
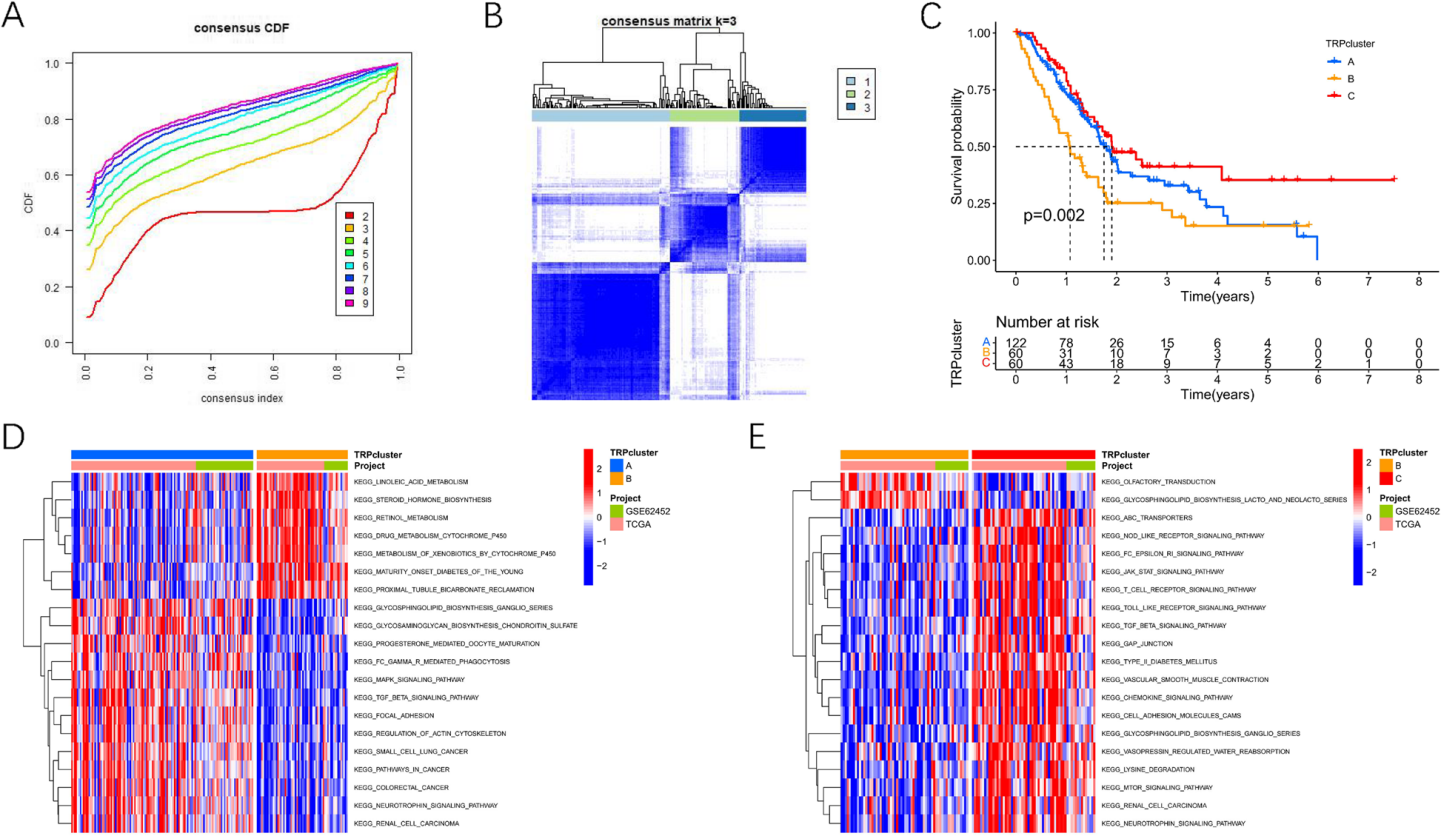
Unsupervised clustering based on 28 TRP-related genes. **A**. Consensus values range from 0 to 1. **B**. Consensus clustering matrix for k = 3. **C**. Kaplan-Meier survival analysis among three clusters. **D**-**E**. GSVA enrichment analysis showing the activation states of biological pathways in distinct clusters, and red represented activated pathways and blue represented inhibited pathways

**Fig. 3:**
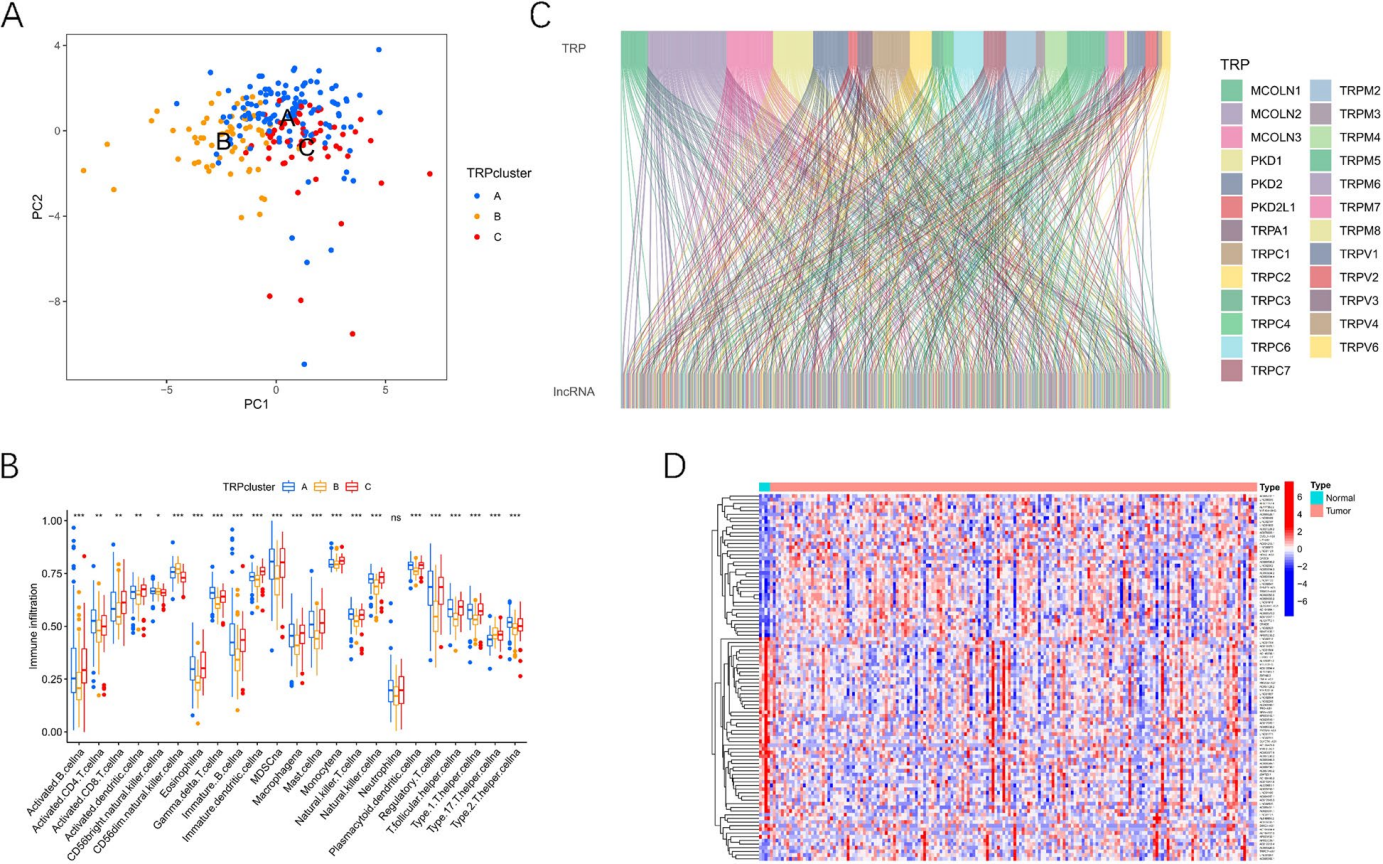
PCA analysis and immune cell infiltration. **A**. Principal components analysis on the basis of 28 TRP family genes. **B**. The difference of immune cell infiltration among three clusters. **C**. Identification of TRP-associated lncRNAs by Pearson correlation analysis. **D**. A heatmap showing TRP-associated lncRNAs with significant differences. (*P < 0.05; **P < 0.01; ***P < 0.001)

Three different subtypes were subsequently identified on the basis of the expression of 28 TRP-related genes with 122 patients in cluster A, 60 patients in cluster B, and 60 patients in cluster C (Figs. 2 A-B). Figure 2 C illustrated remarkable differences in OS among these three clusters (*p <* 0.05). GSVA enrichment analysis helped us understand the biological mechanisms involved in different clusters. As demonstrated in Fig. 2 D, cluster A was highly enriched in carcinogenic signaling pathways including the TGF beta signaling pathway, MAPK signaling pathways, focal adhesion, and pathways in cancer. Cluster C was enriched in immune-related pathways including T cell receptor signaling pathway, Toll-like receptor signaling pathways, JAK/STAT signaling pathway, chemokine signaling pathway, and mTOR signaling pathway (Fig. 2 E). Furthermore, PCA revealed remarkable differences among the three clusters (Fig. 3 A). Following investigations of immune cell infiltration, it was discovered that the abundance level of tumor-infiltrating immune cells in cluster B was considerably lower than that in other clusters (Fig. 3 B), which was linked to poor survival in cluster B. In addition, we also compared the differential expression of TRP-related genes among the three clusters to screen out the relatively important TRP-related genes in each cluster. The result showed that TRPC4, MCOLN3, TRPV5 and TRPV6 were highly expressed in cluster A. And TRPV1, TRPC1, TRPC3, MCOLN1, PKD1, PKD2L1 and TRPV2 were highly expressed in cluster B. Only TRPM4 was highly expressed in cluster C (Supplementary Fig. 5) .

### Identification of TRP-associated lncRNAs with significant prognostic value in pancreatic adenocarcinoma

**Fig. 4:**
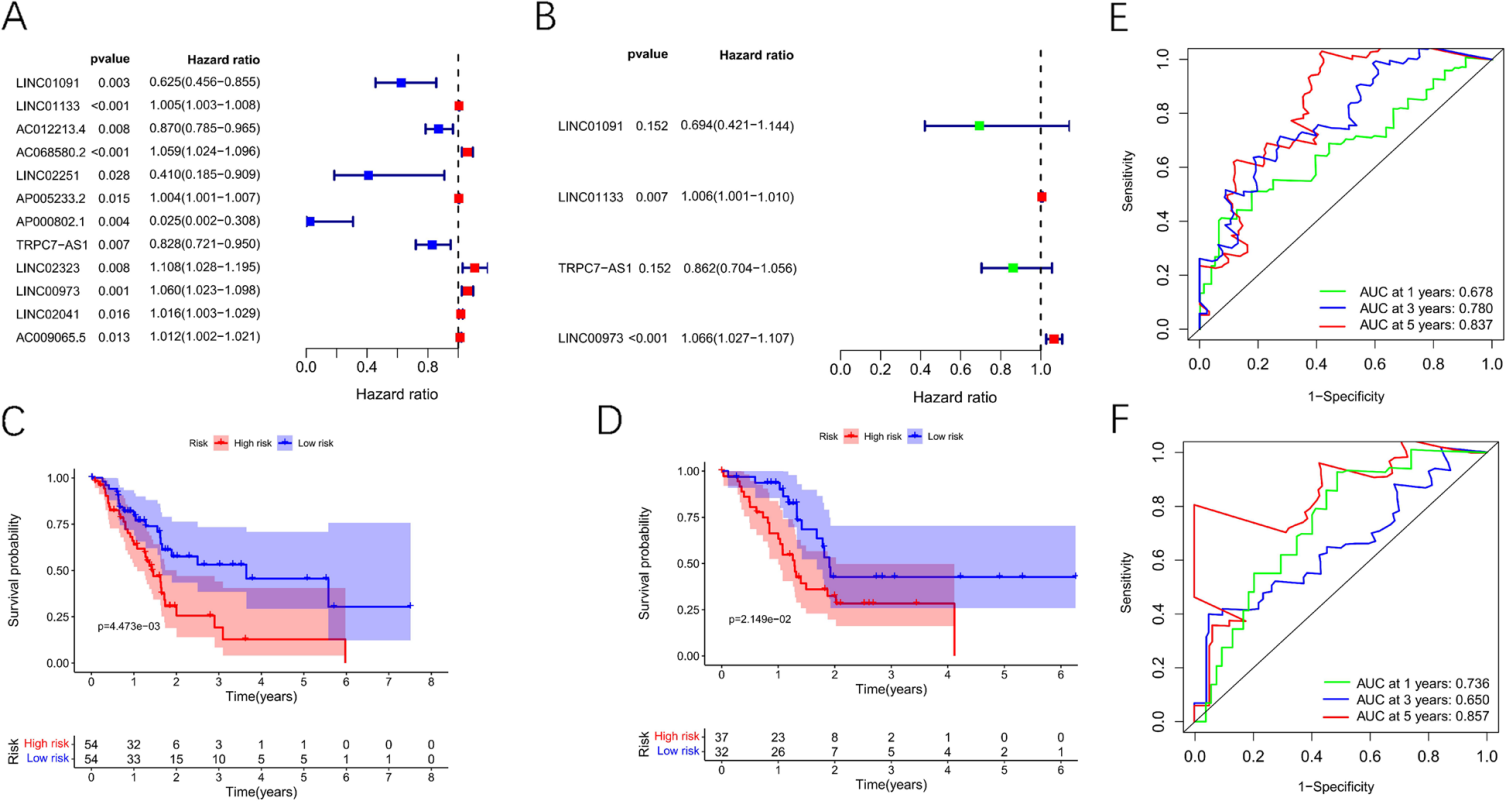
Development of a risk score model based on TRP-associated lncRNAs. **A**-**B**. Univariate Cox analysis and multivariate Cox analysis for TRP-associated lncRNAs. **C**-**D**. Kaplan-Meier survival analysis of high-risk and low-risk group in the training and validation cohorts. **E**-**F**. Receiver operating characteristic (ROC) curves for 1, 3, and 5 years survival in the training and validation cohorts

The Limma R package was performed for the identification of TRP-associated lncRNAs by Pearson correlation analysis. Finally, 345 TRP-associated lncRNAs were used for the following analysis (Fig. 3 C). And the detailed lncRNA-mRNA pairs information was presented in Supplementary Table 1. lncRNAs with significant differences between adjacent normal pancreatic tissues and pancreatic tumor tissues were shown in the heatmap (|logFC| > 2 & FDR < 0.05, Fig. 3 D). Univariate Cox proportional hazards analysis was used to screen 12 TRP-related lncRNAs which were prominently related to patients’ prognosis (*p* < 0.05). As shown in Fig. 4 A, LINC01091, AC012213.4, LINC02251, AP000802.1 and TRPC7 − AS1 were low risk with hazard ration (HR) < 1, whereas LINC01133, AC068580.2, AP005233.2, LINC02323, LINC00973, LINC02041 and AC009065.5 were high risk with hazard ration (HR) > 1. Afterward, a prognostic signature comprising 4 critical lncRNAs, including LINC01091, LINC01133, TRPC7-AS1, and LINC00973, was selected by multivariate Cox analysis in the training set (Fig. 4 B).

### Validation of the 4-TRlncRNAs prognostic signature

On the basis of their median risk level, patients in the training cohort were categorized into high and low-risk groups. The survival curve highlighted, in particular, that PAAD patients in the low-risk group have considerably longer survival times as compared to the high-risk group (Fig. 4 C). Moreover, the AUC of risk score at 1, 3, and 5-years was 0.678, 0.780, and 0.837 respectively, indicating a high predictive value for the prognosis of PAAD patients (Fig. 4 E). Additionally, we also looked into the prognostic model’s predictive values in the validation cohort. The survival curve in the validation cohort demonstrated that PAAD patients in the low-risk group had better survival outcomes than those in the high-risk group, which was consistent with the findings obtained in the training cohort (Fig. 4 D). The respective AUC values in the validation cohort at 1, 3, and 5 years were 0.736, 0.650, and 0.857 (Fig. 4 F).

### Evaluation of the diagnostic efficiency of 4-TRlncRNAs prognostic signature

**Fig. 5:**
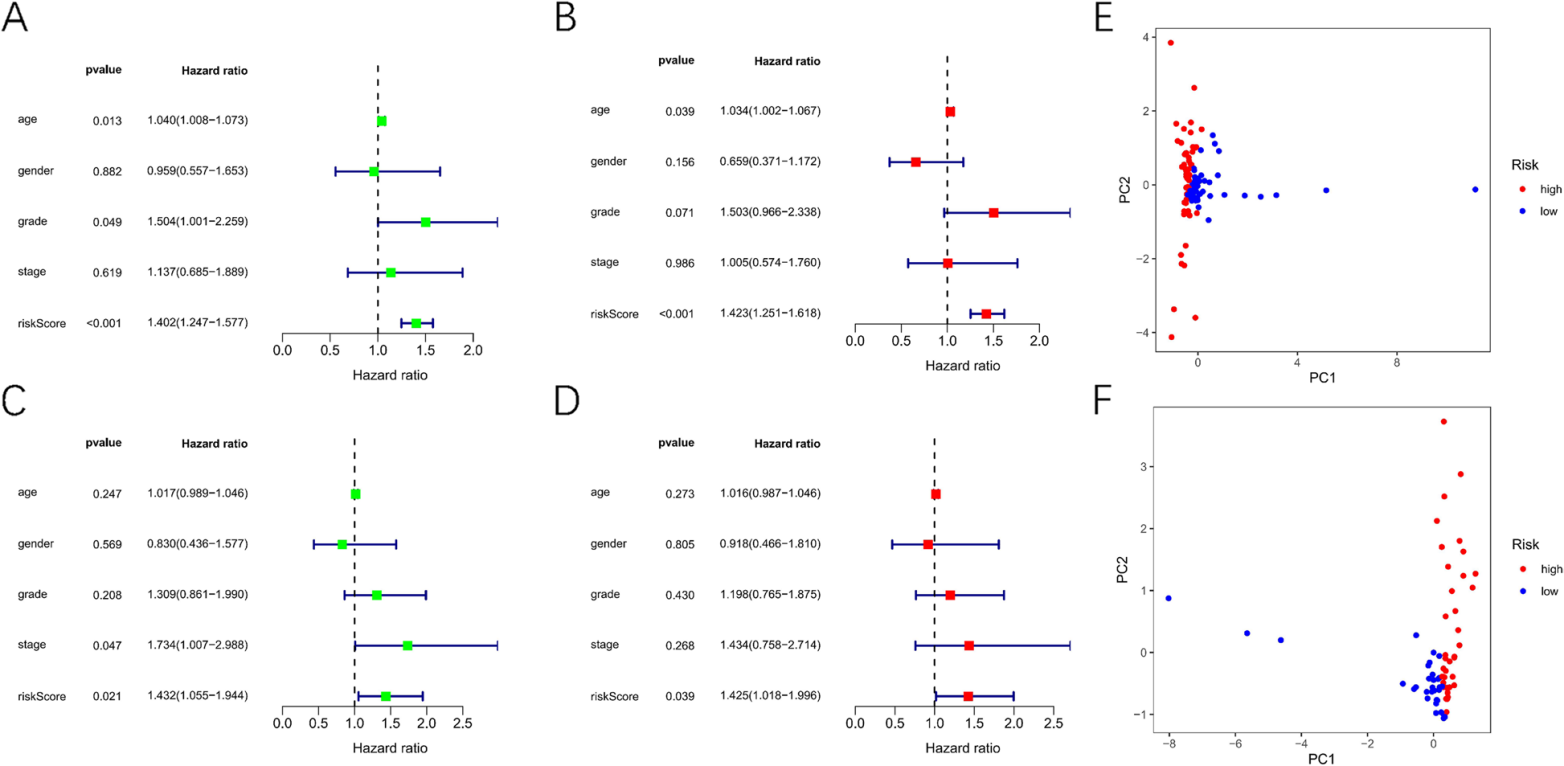
Evaluation of the Diagnostic Efficiency of 4-TRlncRNAs prognostic signature. **A**-**B**. Univariate Cox analysis and multivariate Cox analysis for risk score and clinical features in the training cohort. **C**-**D**. Univariate Cox analysis and multivariate Cox analysis for risk score and clinical features in the validation cohort. **E**-**F**. Principal component analysis between high-risk and low-risk groups in the training and validation cohorts

In addition, we did univariate and multivariate Cox regression analyses for investigating if the 4-TRlncRNAs signature is not dependent on other clinicopathological factors in predicting the survival of PAAD patients. The risk score’s hazard ratio (HR) and 95% CI in the training cohort were 1.402 and 1.247–1.577 (*p <* 0.001) in univariate Cox regression analysis (Fig. 5 A), and 1.423 and 1.251–1.618 (*p <* 0.001) in multivariate Cox regression analysis (Fig. 5 B), showing that 4-TRlncRNAs signature is an independent prognostic factor for PAAD patients. Moreover, findings in the validation cohort verified the signature’s independent prognostic value (Figs. 5 C-D). After dimensionality reduction, we performed PCA on the model genes in the training and validation cohorts based on the high and low-risk groups, and the scatter plot revealed that high and low-risk patients were considerably distinguished, demonstrating that the model genes were the main factor influencing the distribution of high-low risk groups in pancreatic adenocarcinoma (Figs. 5 E-F).

### Establishment and verification of nomogram model for predicting the survival rate of PAAD patients

**Fig. 6:**
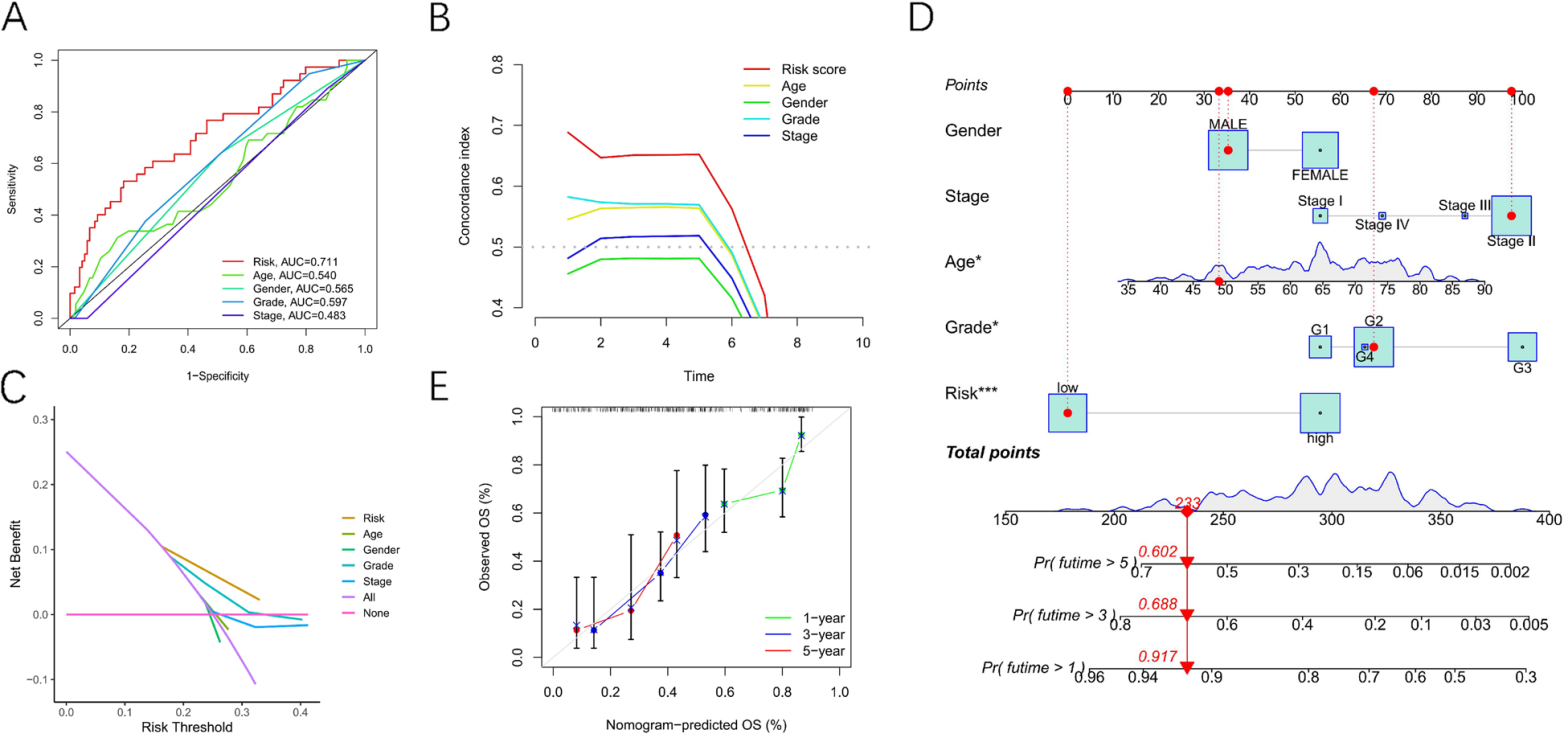
Establishment and verification of a nomogram model. **A**-**C**. ROC curves, C-index and DCA for 4-TRlncRNAs signature and clinicopathological features. **D**. The nomogram consists of clinical characteristics and prognostic signature. E. The nomogram calibration curve is used to predict 1, 3, and 5 years survival rates

**Fig. 7:**
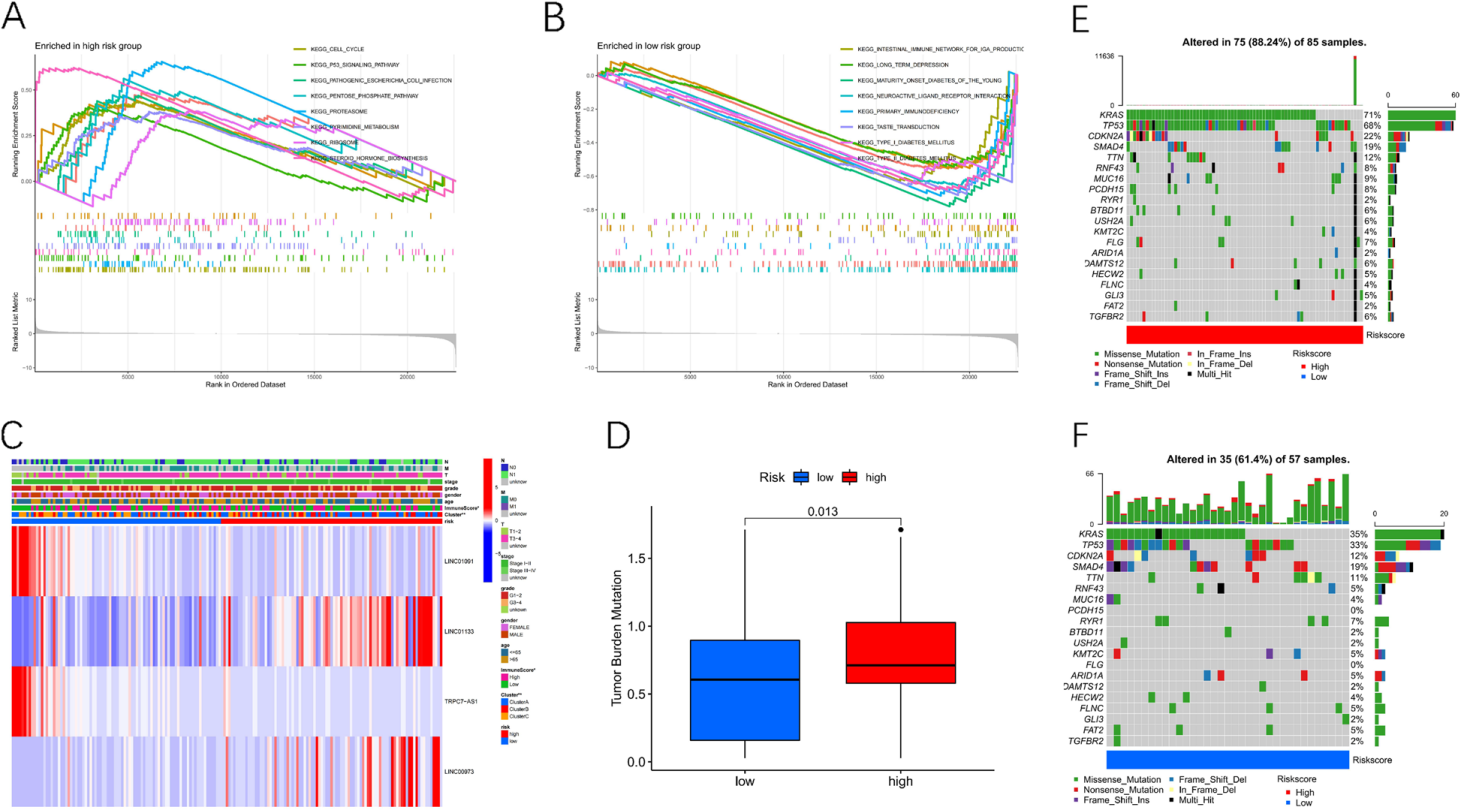
The association between the risk signature with TMB. **A**-**B**. Gene Set Enrichment Analysis for high-risk and low-risk group. **C**. A heatmap depicting 4-TRlncRNAs expression in the high-risk and low-risk group. **D**. The TMB of PC patients in the high-risk and low-risk groups. **E**-**F**. The waterfall plot of tumor somatic mutation in the high-risk and low-risk groups

ROC curves for 4-TRlncRNAs signature and clinicopathological features were created to compare diagnostic effectiveness between the risk score and clinicopathological features, as shown in Fig. 6 A. In PAAD patients, the AUC of 4-TRlncRNAs signature was higher as compared to those of the clinical indexes (AUC = 0.711). The risk score’s C-index was also considerably higher as compared to other clinical features (Fig. 6 B). In clinical decision making, DCA further showed that risk score was a viable prognostic indicator as compared to other variables (Fig. 6 C). After that, we effectively constructed a prognostic nomogram for predicting the 1/3/5-year survival rate. The final OS prediction model incorporated age, gender, grade, stage, and 4-TRlncRNAs (Fig. 6 D). To examine the congruence between actual and expected survival, calibration curves for 1–3-, and 5-year survival were generated, which almost fell on the diagonal of 45°, indicating that the model has good accuracy (Fig. 6 E). Then, using GSEA, it was discovered that cancer-related pathways, such as the cell cycle and the p53 signaling pathway, were considerably enriched in the high-risk group (Figs. 7 A-B).

### The association between the risk signature with TMB

In the high-risk group, LINC01091 and TRPC7 − AS1 had low expression but LINC01133 and LINC00973 had high expression according to the clustering heatmap of the four TRP-associated IncRNAs. Additionally, after comparing high and low-risk groups, there were considerable differences in the immune score and cluster grouping (Fig. 7 C). Moreover, data on somatic mutation were further studied to investigate mutation differences in our risk model. TMB and risk score had a substantial positive link as displayed in Fig. 7 D, with patients in the high-risk group having a higher TMB. Figures 7 E-F depicted the top 20 mutated genes in two risk groups. With an overall mutation rate of 88.24% versus 61.4%, the high-risk group showed a more extensive tumor mutation burden as compared to the low-risk group.

### Relationship between risk score model and immune infiltration

**Fig. 8:**
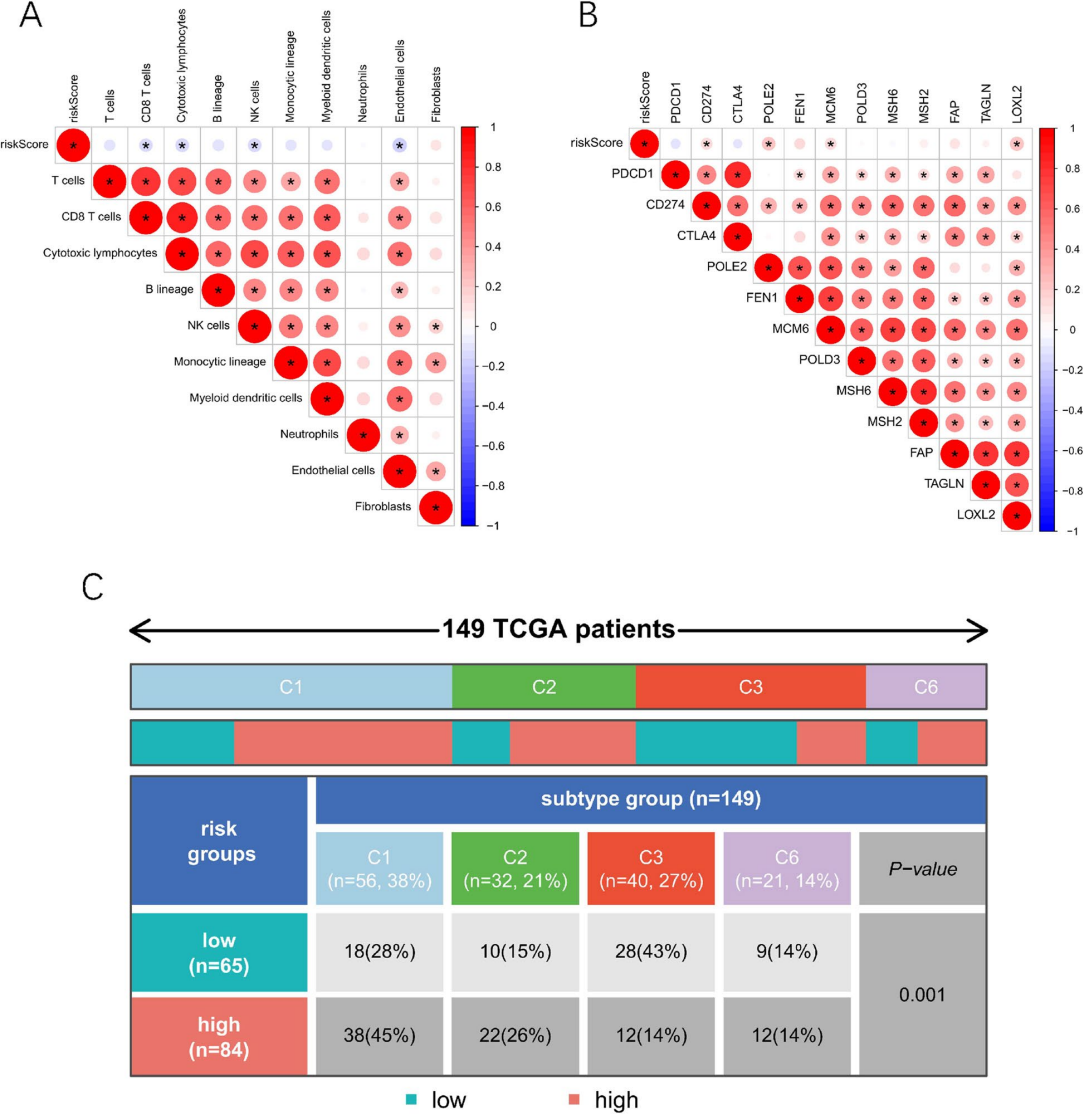
Relationship between risk score model and immune infiltration. **A**. The correlation of riskscore with immune cell infiltration based on MCP-counter algorithm. **B**. The correlation of riskscore with immune checkpoints in PAAD. **C**. Differences in immune subtypes between high and low risk groups

Furthermore, the MCP-counter algorithm revealed that reduced CD8 T cells, NK cells, cytotoxic lymphocytes, and endothelial cells infiltration were associated with a high risk (Fig. 8 A). The expression levels of POLE2, MCM6 CD274, and LOXL2 were found to be higher in the high-risk group (Fig. 8 B), indicating that immune checkpoint inhibitors (ICIs) therapy may benefit high-risk patients more. Moreover, as illustrated in Fig. 8 C, we found considerable changes in the immune subtypes between the high and low-risk groups (*p <* 0.05).

### Validation of TRP-associated lncRNAs in prognostic signature

**Fig. 9:**
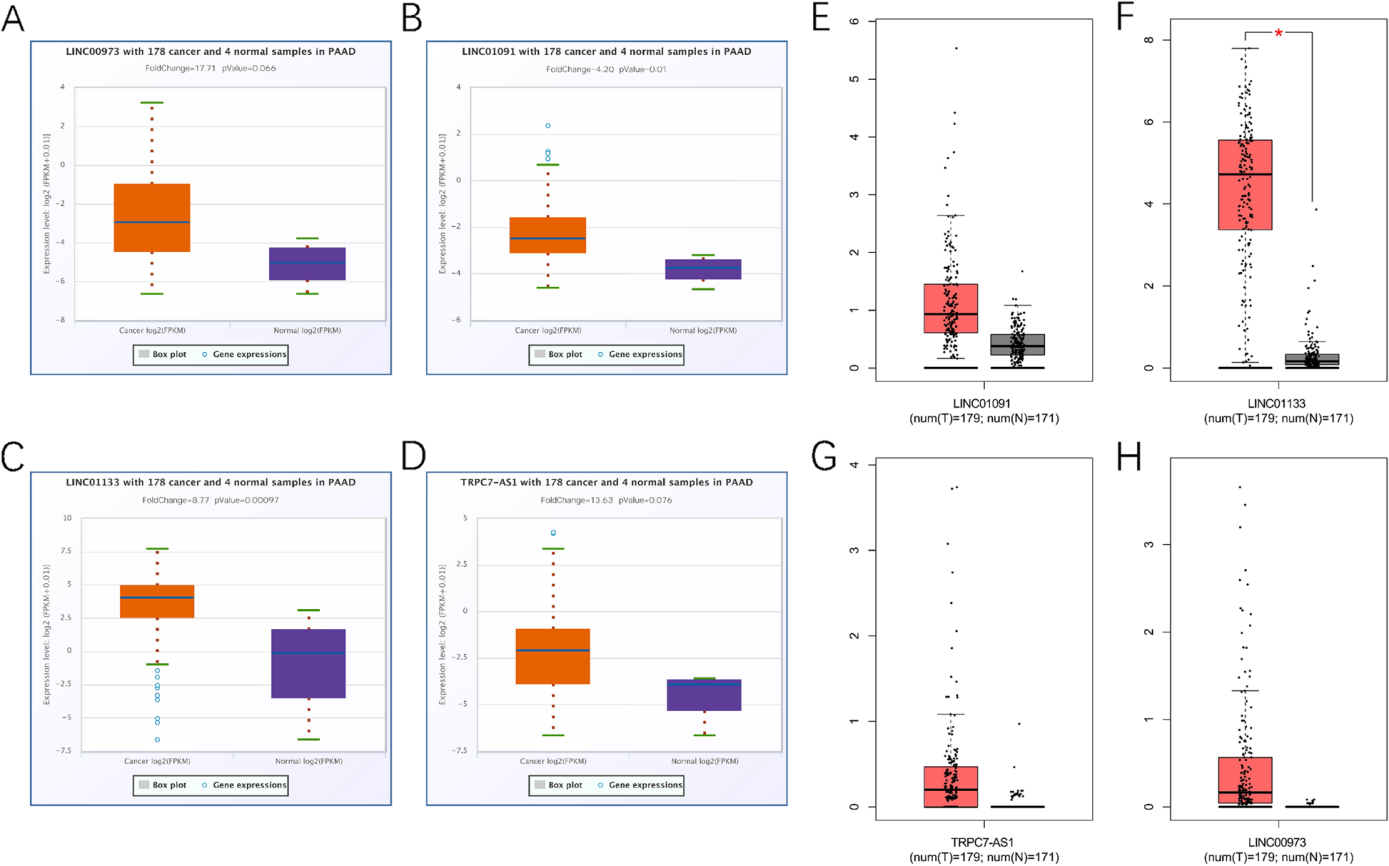
Differential expression of four TRP-associated lncRNAs in public databases. **A**-**D**. The expression of LINC01091, LINC01133, TRPC7-AS1, and LINC00973 in ENCORI database. **E**-**H**. The expression of LINC01091, LINC01133, TRPC7-AS1, and LINC00973 in GEPIA database. (*P < 0.05)

**Fig. 10:**
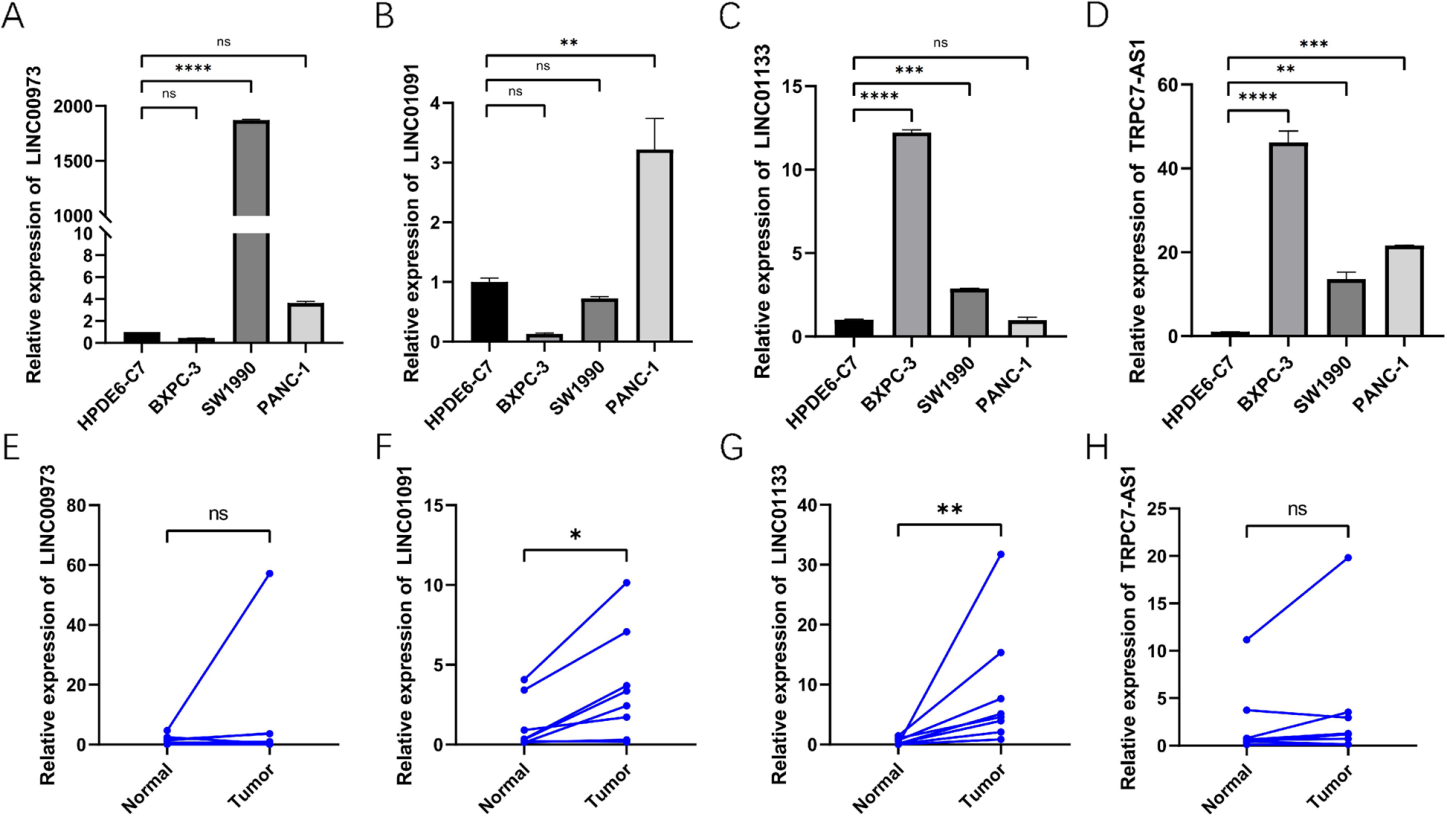
Validation of TRP-associated lncRNAs in pancreatic cancer cells and tissues by qRT-PCR. **A**-**D**. The expression of LINC01091, LINC01133, TRPC7-AS1, and LINC00973 in cell lines. **E**-**H**. The expression of LINC01091, LINC01133, TRPC7-AS1, and LINC00973 in PAAD tissues. (*P < 0.05; **P < 0.01; ***P < 0.001)

Using the ENCORI Pan-Cancer Analysis Platform (https://starbase.sysu.edu.cn/panCancer.php), we looked at the expression of four TRP-associated lncRNAs in normal pancreatic tissues and pancreatic tumor tissues. LINC01091 and LINC01133 expression levels in PAAD tissues were significantly different, as demonstrated in Figs. 9 A-D, whereas TRPC7-AS1 and LINC00973 expression levels were not. However, the ENCORI database contained only four normal pancreatic samples, so we further analyzed the expression of four lncRNAs using the GEPIA website, which incorporated normal pancreas data from the GTEx database. The results showed that only LINC01133 was significantly overexpressed in PAAD tissues, although other lncrnas had no statistical significance, the up-regulation trend was obvious (Figs. 9 E-H). In pancreatic cancer cells and tissues, qRT-PCR indicated the relative expression of LINC01091, LINC01133, TRPC7-AS1, and LINC00973. Figure 10 A-D illustrated the expression levels of four lncRNAs in four cell lines: HPDE6-C7, BXPC-3, SW1990, and PANC-1. In PAAD tissues, LINC01091 and LINC01133 expression levels were significantly different, whereas TRPC7-AS1 and LINC00973 expression levels were not (Fig. 10 E-H). These findings showed that prognostic TRP-associated lncRNAs may play a role in PC tumorigenesis.

## Discussion

Pancreatic adenocarcinoma is a type of gastrointestinal cancer that is highly malignant. PAAD patients have highly invasive and metastatic properties, which lead to a very low overall survival rate [19]. Despite diversified treatment options for pancreatic adenocarcinoma in recent years, the clinical efficacy and overall survival rate have not improved effectively [20]. At present, markers are lacking for predicting the clinical prognosis of PAAD patients effectively. As a result, revealing the molecular mechanism of pancreatic adenocarcinoma occurrence and development as well as exploring potential targets for accurately predicting pancreatic adenocarcinoma prognosis, is of enormous theoretical and clinical importance.

Transient receptor potential (TRP) channels are non-selective cationic channels, that act as tetramers and play a key role in cell perception of environmental changes [21]. In recent years, research has focused on the link between cancer and the TRP channel. A rising number of studies have revealed that TRP channels can interfere with major cancer signaling pathways through Ca2+ signaling dysfunction, causing apoptosis, cell proliferation, gene transcription, and angiogenesis [22, 23]. According to Ding et al. [24], TRPC6 is substantially expressed in oesophageal squamous cell carcinoma (OSCC), and TRPC6 inhibition has the ability to suppress the proliferation of cancer cells and induce G2 /M phase arrest. Another study revealed that TRPM2 can promote migration of gastric cancer, invasion, and tumor growth by regulating PTEN/Akt pathway mediated epithelial-mesenchymal transition (EMT) [25]. According to various studies, TRPC1 expression is also remarkably upregulated in CRC, which promotes metastasis and is linked to a poor prognosis [26**].** Several TRP family compounds have been implicated in pancreatic adenocarcinoma, but the exact functions of TRP family genes in pancreatic adenocarcinoma are yet unknown. TRPM8, which is over-expressed in human pancreatic cancer cell lines, has been found to be required for the proliferation and invasion of pancreatic cancer cells and is closely linked to the sensitivity of gemcitabine, a pancreatic cancer chemotherapy drug [27, 28]. Furthermore, Song et al. [29] discovered that TRPV6 channel protein with high expression level in pancreatic cancer was related to poor prognosis and reduced survival. In a Phase I clinical trial of SOR-C13, a TRPV6 calcium channel inhibitor, two patients having advanced pancreatic cancer indicated a reduction in tumor, along with a 55% reduction in CA19–9 in one patient [30]. However, another study found that low TRPV6 expression was associated with advanced TNM stage, lymph node metastasis and distant metastasis, indicating the anti-oncogenic role of TRPV6 in pancreatic cancer [31]. Thus, TRPV6 has pleiotropic effects in pancreatic cancer, which may depend on the type of cancer and tumor microenvironment. In addition, Lin et al. [32] showed that TRPM2 mutation was associated with the survival time of pancreatic cancer patients, and TRPM2 overexpression could promote PANC-1 cell proliferation and invasion. Therefore, the family of TRP ion channels plays a significant role in pancreatic cancer onset, progression, and chemotherapy resistance. In this study, according to the expression of 28 TRP family genes, three separate subtypes were eventually discovered with the help of unsupervised clustering analysis. In the meantime, cluster B is linked to a poor prognosis and also had the least immune cell infiltration among the three clusters. Additionally, GSVA enrichment analysis further revealed that cluster A was highly enriched in carcinogenic signaling pathways, while cluster C was concentrated in immune-related pathways.

Long non-coding RNAs (lncRNAs) have been implicated in pancreatic cancer invasion, progression, and metastasis [33–35]. Research suggests that lncRNA PSMB8-AS1 has the ability to regulate PD-L1 expression and consequently plays a role in the progression of pancreatic cancer [36]. Moreover, it was discovered by Zhu et al. [37] that lncRNA CRNDE was significantly expressed in pancreatic cancer tissues and enhanced the cancer progression and angiogenesis by regulating the miR-451a/CDKN2D axis. Meanwhile, numerous studies have revealed the involvement of lncRNA in gemcitabine resistance in pancreatic cancer, including PVT1, HIF1A-AS1, UCA1, etc. [38–40]. To study the exact role of TRP-associated lncRNAs in the prognosis of PAAD, we developed a predictive risk model according to prognostic TRP-associated lncRNAs, including LINC01133, LINC01091, TRPC7-AS1, and LINC00973. Research has indicated that LINC01091 expression is lowered in clobetasol propionate-treated skin [41]. An increasing number of reports have revealed subsequently that LINC01133 is deregulated in many human cancers [42, 43]. LINC01133 appears to have a tissue-specific function, serving as either an oncogene or a tumor suppressor gene in various cancer types. Yang et al. [44] reported that low LINC01133 expression level is related to poor prognosis and aggressive tumor phenotype in gastric cancer patients, which could inhibit GC progression and metastasis by regulating the expression of APC and the Wnt/β-catenin pathway. Though, it has been reported that LINC01133 has a carcinogenic role as it activates CCNG1 in pancreatic cancer [45]. Likewise, LINC01133 has also been found to play a tumorigenic role in HCC as it promotes the carcinogenic PI3K/AKT signaling pathway [46]. A novel lncRNA, TRPC7-AS1, positioned at chromosome 5q31.1, was originally known as a potential oncogene in HCC tissues. Moreover, the m6A level of TRPC7-AS1 in HepG2 cells was considerably lower compared to that in LO2 cells [47]. Research has suggested that high LINC00973 is closely correlated with chemotherapy resistance in colorectal cancer [48, 49]. Additionally, LINC00973 was involved in cancer immunosuppression by modulating SIGLEC-15 in ccRCC [50]. Additional analyses showed that 4 TRP-associated lncRNAs have the potential to predict survival in PAAD patients independently of other clinicopathological factors and diagnostic efficiency of risk score was greatly enhanced as compared to other clinical indicators. Furthermore, a nomogram based on gender, age, grade, stage, and risk score may effectively predict PAAD patients’ prognosis. GSEA also revealed that cancer-related pathways, including the p53 signaling pathway and cell cycle, were considerably enriched in high-risk patients. Furthermore, after comparing high and low-risk groups, there were remarkable differences in immune scores and cluster grouping. Furthermore, the MCP-counter algorithm revealed that lower levels of T cells, CD8 cytotoxic lymphocytes, NK cells, and endothelial cells infiltration are associated with high risk. CD274, POLE2, MCM6, and LOXL2 expression levels were all elevated in the high-risk group. TMB level was also greatly increased in high-risk patients, which indicated that immune checkpoint inhibitors (ICIs) therapy may benefit them more.

## Conclusion

Firstly, we used TRP family genes to categorize PAAD patients into three subgroups. Then, using TRP-associated lncRNAs, we constructed a PAAD-related predictive risk model that could reliably predict the prognosis of PAAD patients. Finally, we looked into the link between the prognostic model and the immunological microenvironment. Our research will be useful in guiding treatment and formulating a precise treatment plan. However, there are a few potential flaws in our research. Because all of the expression matrix and clinical data were obtained retrospectively from public sources, our findings are needed to be further verified in well-designed prospective multicenter trials. Besides, further molecular investigations will be needed in the future to validate the function of these TRP-associated lncRNAs. In addition, the regulatory mechanism of TRP-related lncRNA-mRNAs at the cellular level also needs to be further explored.

## Supplementary Information

**Additional file 1.**

## Data Availability

The datasets downloaded for supporting the results of this article are publicly available from TCGA (https://portal.gdc.cancer.gov/) and GEO (https://www.ncbi.nlm.nih.gov/geo/).
