# Supplementary material for: Molecular subtyping based on TRP family and prognostic assessment for TRP-associated lncRNAs in pancreatic adenocarcinoma
Source: BMC Gastroenterol. 2022 Nov 12;22:454. doi: 10.1186/s12876-022-02552-y (PMC9652922; doi:10.1186/s12876-022-02552-y)
Supplement: Supplementary file 1 — Additional file 1. [file 12876_2022_2552_MOESM1_ESM.docx]

**Supplementary Figure 1.** Differential expression of TRP-related genes in pancreatic adenocarcinoma by GEPIA database.

**
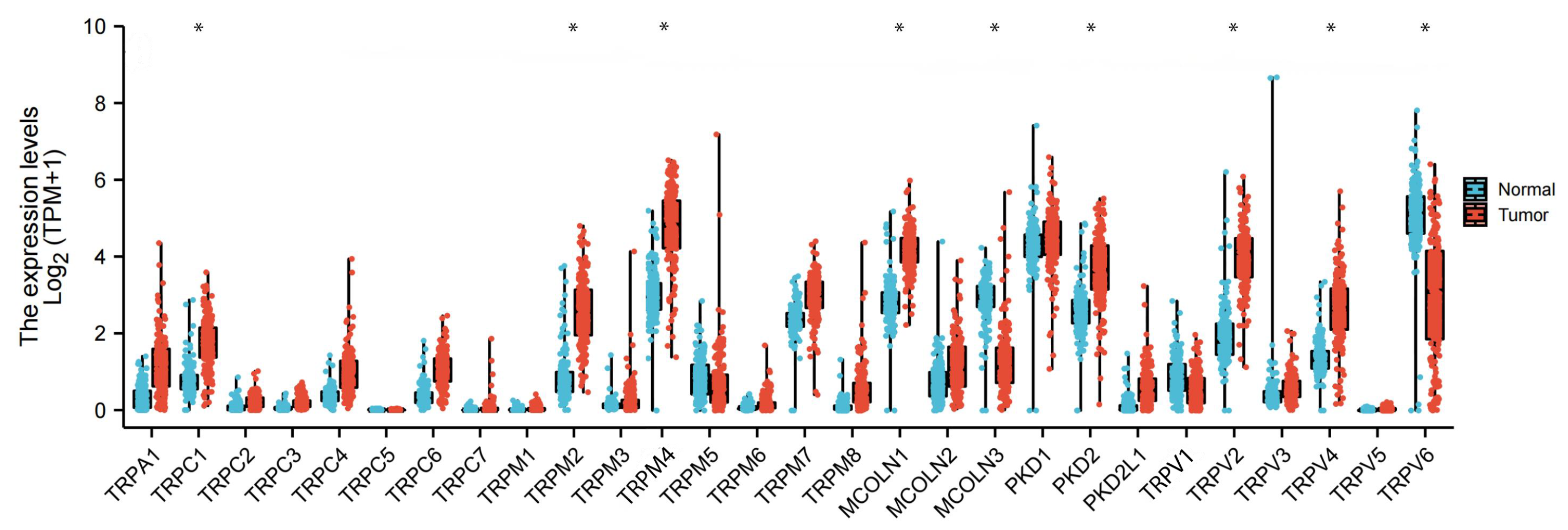
**

**Supplementary Figure 2.** The expression of TRP family genes in pancreatic cancer cell lines via CCLE database.

**
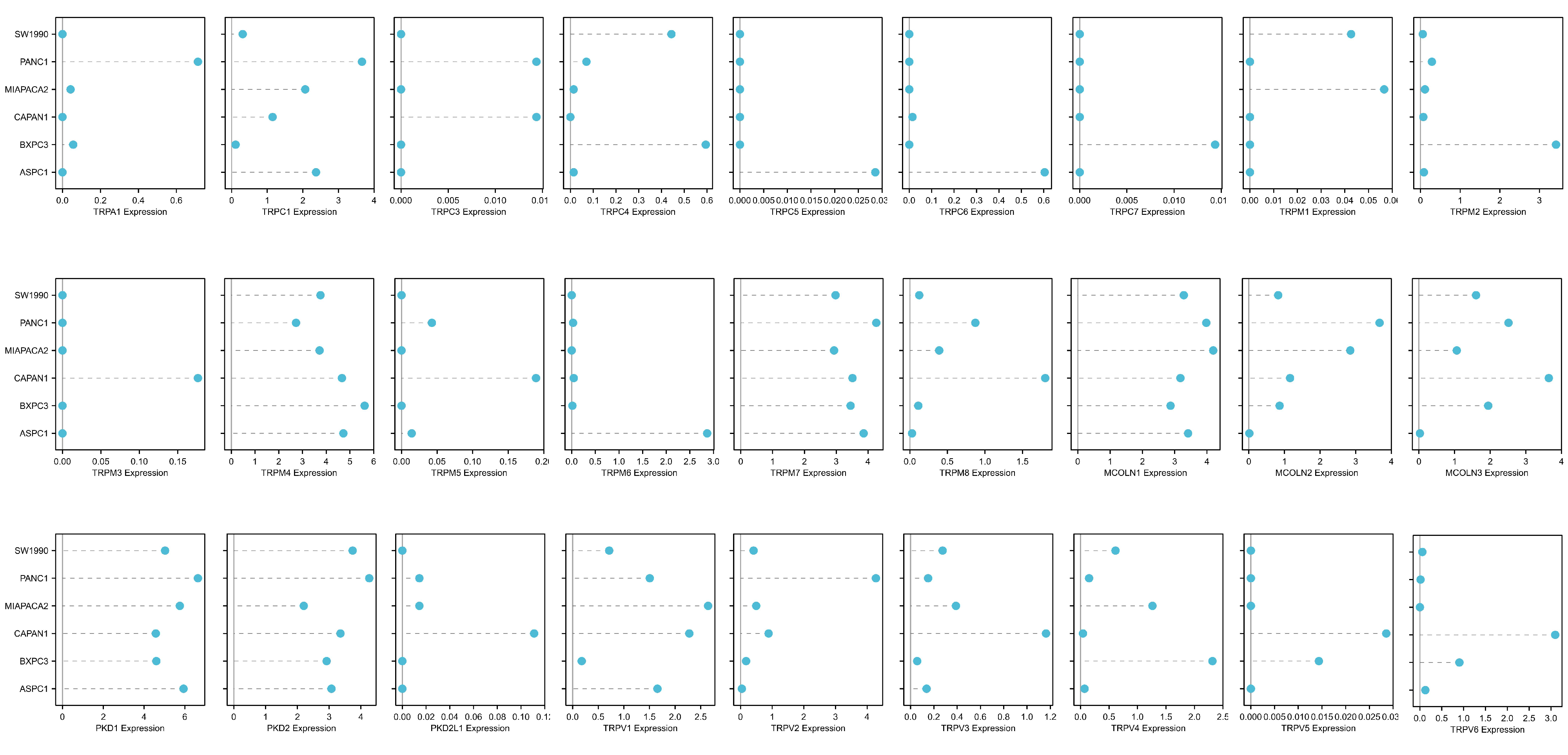
**

**Supplementary Figure 3.** The prognostic value of TRP family genes in pancreatic adenocarcinoma using KM-plotter database.

**
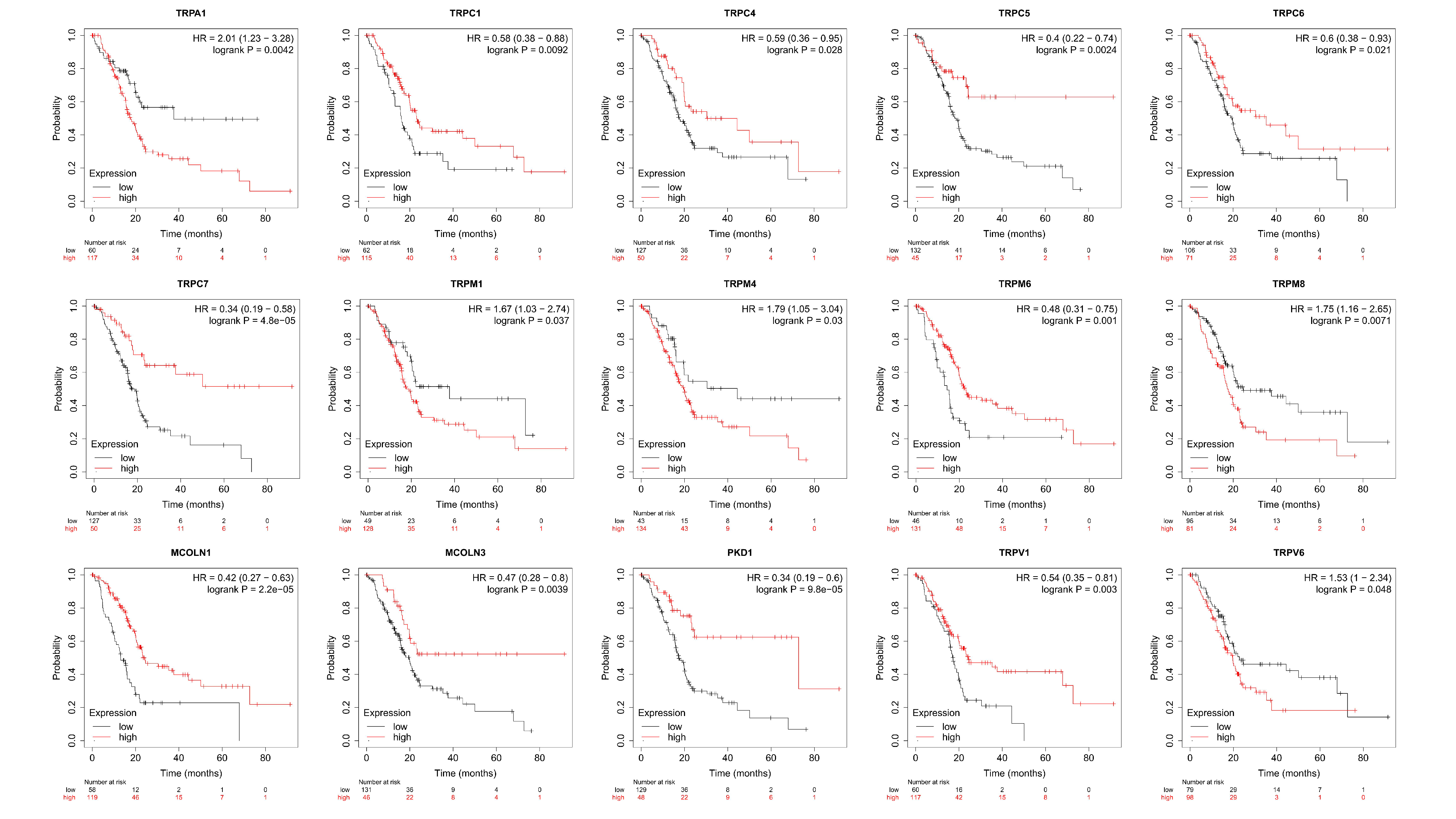
**

**Supplementary Figure 4.** The PPI network of TRP family genes analysed by STRING website.

**
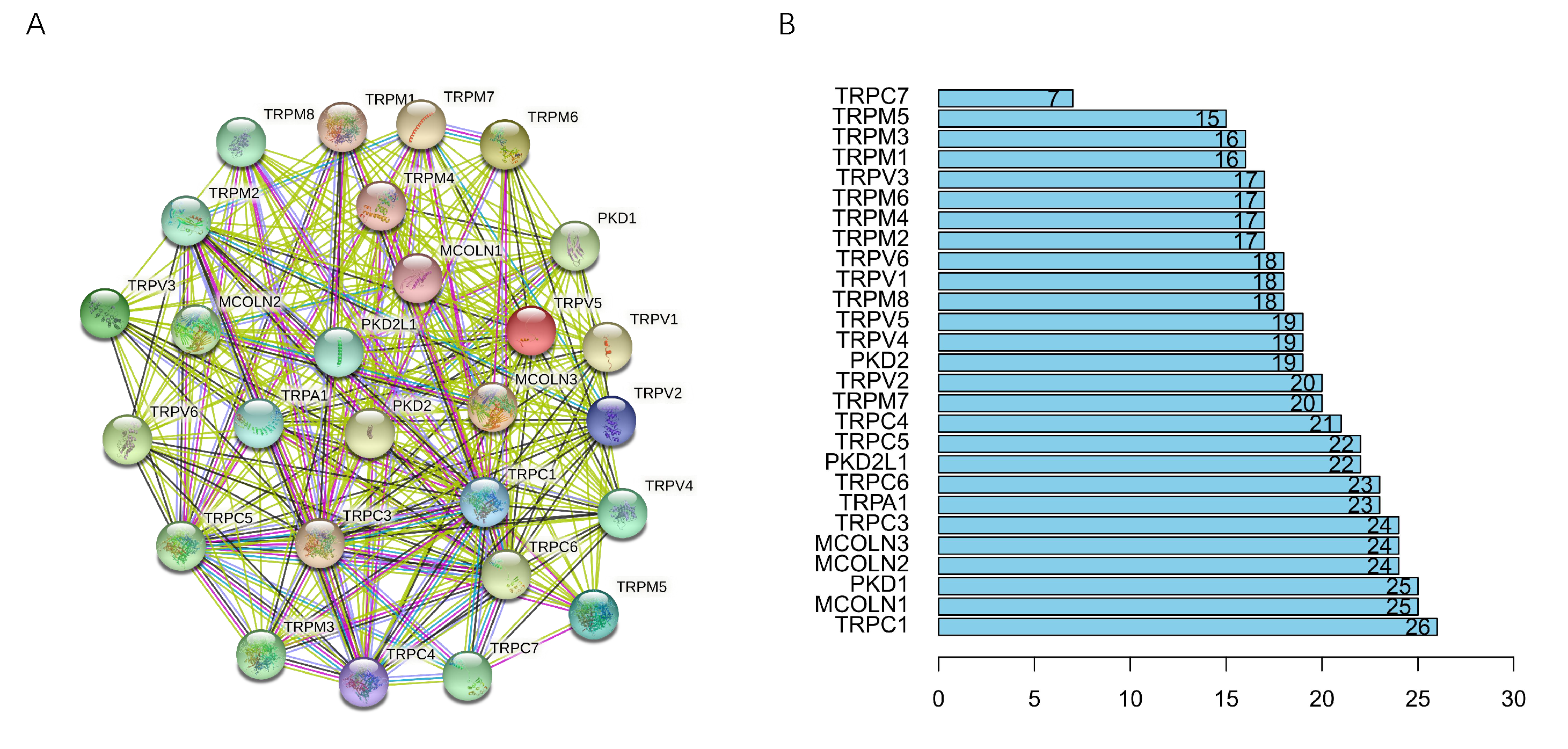
**

**Supplementary Figure 5.** The expression of TRP family genes among three clusters.

**
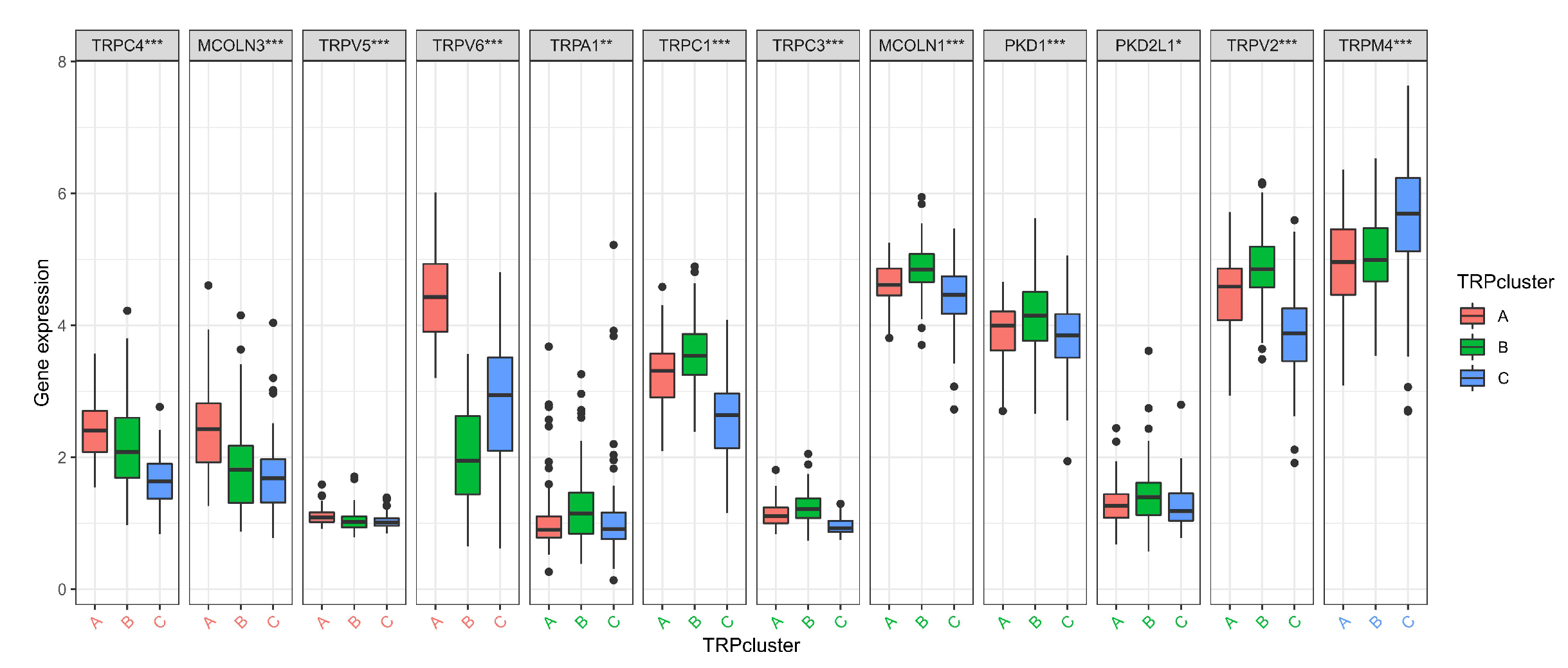
**

**Supplementary Table 1. 345 lncRNAs co-expressed with TRP family genes.**

| TRP | lncRNA | cor | pvalue | Regulation |
| --- | --- | --- | --- | --- |
| TRPM8 | LINC01843 | 0.413190105 | 9.92E-09 | postive |
| TRPV6 | NFIA-AS2 | 0.413470913 | 9.68E-09 | postive |
| TRPC4 | LINC01091 | 0.648212767 | 1.36E-22 | postive |
| TRPC7 | LINC01091 | 0.725973241 | 2.00E-30 | postive |
| TRPM3 | LINC01091 | 0.408426437 | 1.52E-08 | postive |
| MCOLN3 | LINC01091 | 0.531229683 | 2.37E-14 | postive |
| TRPV6 | AC010136.1 | 0.50047861 | 1.13E-12 | postive |
| MCOLN2 | AC243960.1 | 0.812490958 | 4.19E-43 | postive |
| MCOLN2 | AC015911.6 | 0.478574492 | 1.41E-11 | postive |
| TRPC1 | FLG-AS1 | 0.401227493 | 2.85E-08 | postive |
| TRPC4 | FLG-AS1 | 0.527658855 | 3.79E-14 | postive |
| TRPC7 | FLG-AS1 | 0.501501134 | 1.00E-12 | postive |
| TRPM3 | FLG-AS1 | 0.524357389 | 5.82E-14 | postive |
| MCOLN1 | FLG-AS1 | 0.488673827 | 4.51E-12 | postive |
| TRPM4 | PCAT7 | 0.420698895 | 5.01E-09 | postive |
| TRPA1 | AC108860.2 | 0.472171531 | 2.86E-11 | postive |
| TRPC7 | AC005281.1 | 0.617619358 | 4.21E-20 | postive |
| TRPM5 | AC005281.1 | 0.558174347 | 5.74E-16 | postive |
| MCOLN3 | AC005281.1 | 0.607619515 | 2.41E-19 | postive |
| PKD1 | AC005281.1 | 0.57173518 | 7.74E-17 | postive |
| TRPA1 | LINC02489 | 0.578969492 | 2.56E-17 | postive |
| MCOLN2 | AC006946.3 | 0.611521903 | 1.23E-19 | postive |
| MCOLN2 | AC136475.8 | 0.426223075 | 3.00E-09 | postive |
| PKD1 | LINC01284 | 0.489404757 | 4.14E-12 | postive |
| TRPV6 | AC022075.1 | 0.435256757 | 1.27E-09 | postive |
| TRPV1 | AC009087.1 | 0.410460924 | 1.27E-08 | postive |
| TRPC6 | PDCD4-AS1 | 0.404310516 | 2.18E-08 | postive |
| MCOLN2 | PDCD4-AS1 | 0.662673012 | 7.14E-24 | postive |
| MCOLN2 | LINC02539 | 0.407420532 | 1.66E-08 | postive |
| TRPC1 | COL4A2-AS1 | 0.400484634 | 3.04E-08 | postive |
| TRPC6 | COL4A2-AS1 | 0.433284921 | 1.53E-09 | postive |
| PKD2 | COL4A2-AS1 | 0.42823164 | 2.48E-09 | postive |
| MCOLN2 | AC012020.1 | 0.655712866 | 3.01E-23 | postive |
| TRPM4 | AC007255.1 | 0.572705344 | 6.68E-17 | postive |
| TRPM4 | AL109976.1 | 0.420833319 | 4.95E-09 | postive |
| PKD2 | LINC01354 | 0.48773376 | 5.02E-12 | postive |
| TRPM7 | AC116158.1 | 0.41783097 | 6.52E-09 | postive |
| TRPV1 | AC116158.1 | 0.440571383 | 7.54E-10 | postive |
| TRPC6 | PCAT19 | 0.500355613 | 1.15E-12 | postive |
| MCOLN2 | PCAT19 | 0.473269728 | 2.54E-11 | postive |
| PKD2 | PCAT19 | 0.412228251 | 1.08E-08 | postive |
| MCOLN2 | EML4-AS1 | 0.780051375 | 1.11E-37 | postive |
| TRPM2 | AL121985.1 | 0.441093473 | 7.17E-10 | postive |
| MCOLN2 | AL121985.1 | 0.809879493 | 1.25E-42 | postive |
| TRPC1 | PPM1F-AS1 | 0.456723897 | 1.48E-10 | postive |
| MCOLN1 | PPM1F-AS1 | 0.410739345 | 1.24E-08 | postive |
| PKD1 | PPM1F-AS1 | 0.507088008 | 5.09E-13 | postive |
| TRPM4 | TONSL-AS1 | 0.444599718 | 5.06E-10 | postive |
| TRPM5 | AC008629.1 | 0.507969469 | 4.57E-13 | postive |
| MCOLN3 | AC008629.1 | 0.540387246 | 6.95E-15 | postive |
| TRPC1 | LRRK2-DT | 0.466088013 | 5.51E-11 | postive |
| TRPC6 | LRRK2-DT | 0.467815755 | 4.58E-11 | postive |
| MCOLN2 | LRRK2-DT | 0.607034143 | 2.66E-19 | postive |
| PKD2 | LRRK2-DT | 0.480388177 | 1.15E-11 | postive |
| TRPM4 | LINC01133 | 0.439855833 | 8.09E-10 | postive |
| TRPM5 | LINC02139 | 0.495827381 | 1.96E-12 | postive |
| MCOLN3 | LINC02139 | 0.458140533 | 1.27E-10 | postive |
| TRPC6 | CARD8-AS1 | 0.502421091 | 8.96E-13 | postive |
| TRPM2 | CARD8-AS1 | 0.500825396 | 1.08E-12 | postive |
| TRPM7 | CARD8-AS1 | 0.498684397 | 1.40E-12 | postive |
| MCOLN2 | CARD8-AS1 | 0.77716464 | 3.06E-37 | postive |
| PKD2 | CARD8-AS1 | 0.478979893 | 1.35E-11 | postive |
| TRPV2 | CARD8-AS1 | 0.413287222 | 9.84E-09 | postive |
| TRPM5 | MEG3 | 0.908652657 | 1.16E-68 | postive |
| MCOLN3 | MEG3 | 0.799924089 | 6.94E-41 | postive |
| PKD1 | MEG3 | 0.487171493 | 5.35E-12 | postive |
| TRPV4 | AL513327.2 | 0.418189756 | 6.31E-09 | postive |
| TRPM5 | ZMIZ1-AS1 | 0.48541933 | 6.54E-12 | postive |
| TRPC1 | AL606834.2 | 0.448866291 | 3.30E-10 | postive |
| TRPM5 | AL606834.2 | 0.529642672 | 2.92E-14 | postive |
| TRPM6 | AL606834.2 | 0.413874016 | 9.33E-09 | postive |
| MCOLN2 | AL606834.2 | 0.55411433 | 1.03E-15 | postive |
| MCOLN3 | AL606834.2 | 0.441012315 | 7.22E-10 | postive |
| MCOLN2 | AC007036.1 | 0.550309745 | 1.76E-15 | postive |
| TRPM5 | AP000255.1 | 0.421587533 | 4.61E-09 | postive |
| MCOLN3 | AP000255.1 | 0.410132988 | 1.30E-08 | postive |
| TRPM4 | AC008870.3 | 0.517993308 | 1.31E-13 | postive |
| TRPM5 | LINC01978 | 0.712203009 | 7.52E-29 | postive |
| MCOLN3 | LINC01978 | 0.504120993 | 7.30E-13 | postive |
| TRPC1 | AP003559.1 | 0.456532658 | 1.51E-10 | postive |
| TRPC7 | AP003559.1 | 0.476668355 | 1.74E-11 | postive |
| MCOLN1 | AP003559.1 | 0.591889655 | 3.30E-18 | postive |
| PKD1 | AP003559.1 | 0.462158959 | 8.36E-11 | postive |
| TRPC2 | AC020658.5 | 0.429351191 | 2.23E-09 | postive |
| MCOLN2 | AC020658.5 | 0.416278433 | 7.51E-09 | postive |
| TRPV1 | AC092802.2 | 0.480860666 | 1.09E-11 | postive |
| TRPC1 | AC093010.2 | 0.5271507 | 4.05E-14 | postive |
| TRPC6 | AC093010.2 | 0.471017369 | 3.24E-11 | postive |
| TRPM7 | AC093010.2 | 0.427141514 | 2.75E-09 | postive |
| PKD2 | AC093010.2 | 0.61368564 | 8.42E-20 | postive |
| MCOLN2 | AC104653.1 | 0.744342253 | 1.10E-32 | postive |
| TRPM4 | BX470102.1 | 0.488354667 | 4.68E-12 | postive |
| MCOLN2 | AC004687.1 | 0.595547717 | 1.82E-18 | postive |
| MCOLN2 | AC025279.1 | 0.683223993 | 8.09E-26 | postive |
| TRPC1 | CHROMR | 0.621659613 | 2.05E-20 | postive |
| TRPM7 | CHROMR | 0.455607532 | 1.66E-10 | postive |
| PKD1 | CHROMR | 0.40661846 | 1.78E-08 | postive |
| PKD2 | CHROMR | 0.562444526 | 3.08E-16 | postive |
| TRPC2 | AC138207.5 | 0.550380663 | 1.74E-15 | postive |
| TRPC6 | AC138207.5 | 0.442990758 | 5.94E-10 | postive |
| TRPM2 | AC138207.5 | 0.494371043 | 2.33E-12 | postive |
| TRPV2 | AC138207.5 | 0.422598022 | 4.20E-09 | postive |
| TRPM5 | AL645608.2 | 0.811469882 | 6.43E-43 | postive |
| MCOLN3 | AL645608.2 | 0.646481441 | 1.91E-22 | postive |
| TRPM5 | LINC01833 | 0.615108769 | 6.56E-20 | postive |
| MCOLN3 | LINC01833 | 0.49024827 | 3.76E-12 | postive |
| TRPM4 | AC024941.2 | 0.46366368 | 7.13E-11 | postive |
| MCOLN2 | SNHG26 | 0.486304566 | 5.91E-12 | postive |
| MCOLN3 | TMEM254-AS1 | 0.476777753 | 1.72E-11 | postive |
| TRPC1 | SUGT1P4-STRA6LP | 0.516671698 | 1.55E-13 | postive |
| TRPC4 | SUGT1P4-STRA6LP | 0.499239397 | 1.31E-12 | postive |
| PKD1 | SUGT1P4-STRA6LP | 0.582536871 | 1.47E-17 | postive |
| TRPM5 | AC080013.5 | 0.653032067 | 5.18E-23 | postive |
| MCOLN3 | AC080013.5 | 0.700328689 | 1.45E-27 | postive |
| TRPM4 | CRNDE | 0.500410399 | 1.14E-12 | postive |
| TRPM5 | AL132800.1 | 0.545735927 | 3.33E-15 | postive |
| MCOLN3 | AL132800.1 | 0.535291581 | 1.38E-14 | postive |
| TRPC4 | LINC00589 | 0.473312143 | 2.52E-11 | postive |
| MCOLN2 | AC067945.2 | 0.79042961 | 2.61E-39 | postive |
| MCOLN2 | LINC00852 | 0.571324842 | 8.23E-17 | postive |
| MCOLN2 | DBH-AS1 | 0.609109742 | 1.86E-19 | postive |
| MCOLN2 | AC104653.2 | 0.687773205 | 2.85E-26 | postive |
| TRPC3 | AC087627.1 | 0.412927481 | 1.02E-08 | postive |
| TRPC2 | AC006272.1 | 0.500930296 | 1.07E-12 | postive |
| PKD2L1 | AC006272.1 | 0.476560521 | 1.77E-11 | postive |
| MCOLN3 | LINC01121 | 0.409488436 | 1.38E-08 | postive |
| TRPC3 | AC007376.2 | 0.411269712 | 1.18E-08 | postive |
| PKD2 | AC007376.2 | 0.429770405 | 2.14E-09 | postive |
| TRPM5 | AP000345.2 | 0.434601826 | 1.35E-09 | postive |
| TRPC1 | FGF14-AS2 | 0.484605324 | 7.17E-12 | postive |
| TRPC6 | FGF14-AS2 | 0.418861405 | 5.93E-09 | postive |
| TRPC7 | FGF14-AS2 | 0.564316358 | 2.34E-16 | postive |
| TRPM4 | FGF14-AS2 | -0.404720235 | 2.10E-08 | negative |
| TRPM5 | FGF14-AS2 | 0.48096702 | 1.08E-11 | postive |
| MCOLN1 | FGF14-AS2 | 0.569381626 | 1.10E-16 | postive |
| MCOLN3 | FGF14-AS2 | 0.627059555 | 7.68E-21 | postive |
| PKD1 | FGF14-AS2 | 0.559417188 | 4.79E-16 | postive |
| MCOLN2 | AC027097.2 | 0.786064538 | 1.30E-38 | postive |
| PKD1 | AC002480.2 | 0.421334744 | 4.72E-09 | postive |
| TRPM5 | AC010247.2 | 0.936734563 | 3.73E-82 | postive |
| MCOLN3 | AC010247.2 | 0.756127661 | 3.09E-34 | postive |
| TRPM5 | AC010809.2 | 0.420504885 | 5.10E-09 | postive |
| MCOLN3 | AC010809.2 | 0.400665239 | 2.99E-08 | postive |
| TRPV1 | AC010809.2 | 0.471506464 | 3.07E-11 | postive |
| TRPC6 | AL590787.1 | 0.456074614 | 1.58E-10 | postive |
| TRPM5 | AL590787.1 | 0.407506466 | 1.65E-08 | postive |
| MCOLN1 | AL590787.1 | 0.494307275 | 2.34E-12 | postive |
| MCOLN3 | AL590787.1 | 0.627348891 | 7.28E-21 | postive |
| TRPM2 | LINC01480 | 0.414726015 | 8.64E-09 | postive |
| MCOLN2 | LINC01480 | 0.779440458 | 1.38E-37 | postive |
| TRPC2 | AC004865.2 | 0.405409639 | 1.98E-08 | postive |
| TRPM2 | AC004865.2 | 0.430580809 | 1.98E-09 | postive |
| MCOLN2 | AC004865.2 | 0.715376016 | 3.32E-29 | postive |
| TRPM2 | AC009119.1 | 0.42040857 | 5.14E-09 | postive |
| MCOLN2 | AC009119.1 | 0.614333862 | 7.52E-20 | postive |
| TRPC1 | AC068473.3 | 0.554449777 | 9.80E-16 | postive |
| TRPC3 | AC068473.3 | 0.40098282 | 2.91E-08 | postive |
| PKD2 | AC068473.3 | 0.533500398 | 1.76E-14 | postive |
| TRPC1 | MIR503HG | 0.424511441 | 3.52E-09 | postive |
| TRPC2 | MIR503HG | 0.51601895 | 1.68E-13 | postive |
| MCOLN2 | CEP250-AS1 | 0.837295255 | 5.08E-48 | postive |
| MCOLN2 | AC007546.1 | 0.613431909 | 8.81E-20 | postive |
| TRPM8 | AC080037.2 | 0.469969124 | 3.63E-11 | postive |
| MCOLN2 | AC079921.2 | 0.540785304 | 6.58E-15 | postive |
| TRPC1 | AC093278.2 | 0.507209824 | 5.01E-13 | postive |
| TRPC6 | AC093278.2 | 0.66649182 | 3.19E-24 | postive |
| TRPM2 | AC093278.2 | 0.404328415 | 2.18E-08 | postive |
| TRPM6 | AC093278.2 | 0.408234266 | 1.54E-08 | postive |
| TRPM7 | AC093278.2 | 0.476552035 | 1.77E-11 | postive |
| MCOLN2 | AC093278.2 | 0.532025605 | 2.14E-14 | postive |
| PKD2 | AC093278.2 | 0.569193649 | 1.13E-16 | postive |
| TRPC7 | ITGA9-AS1 | 0.40381 | 2.28E-08 | postive |
| TRPM5 | ITGA9-AS1 | 0.598905488 | 1.04E-18 | postive |
| MCOLN3 | ITGA9-AS1 | 0.593779023 | 2.43E-18 | postive |
| TRPC4 | AC012213.4 | 0.445664653 | 4.55E-10 | postive |
| TRPC7 | AC012213.4 | 0.640870858 | 5.71E-22 | postive |
| TRPM3 | AC012213.4 | 0.459614402 | 1.09E-10 | postive |
| TRPM5 | AC012213.4 | 0.494587998 | 2.27E-12 | postive |
| MCOLN1 | AC012213.4 | 0.515390788 | 1.82E-13 | postive |
| MCOLN3 | AC012213.4 | 0.816409021 | 7.86E-44 | postive |
| PKD1 | AC012213.4 | 0.444800853 | 4.96E-10 | postive |
| TRPM2 | AC008972.2 | 0.53509206 | 1.42E-14 | postive |
| MCOLN2 | AC008972.2 | 0.424027387 | 3.68E-09 | postive |
| TRPC3 | AL031985.3 | 0.40395351 | 2.25E-08 | postive |
| TRPC6 | AL031985.3 | 0.457026498 | 1.43E-10 | postive |
| TRPM2 | AL031985.3 | 0.490675794 | 3.58E-12 | postive |
| TRPM7 | AL031985.3 | 0.418942932 | 5.89E-09 | postive |
| PKD2 | AL031985.3 | 0.471163947 | 3.19E-11 | postive |
| TRPV2 | AL031985.3 | 0.458034057 | 1.29E-10 | postive |
| TRPM5 | LINC02044 | 0.413708141 | 9.47E-09 | postive |
| MCOLN3 | LINC02044 | 0.536833839 | 1.12E-14 | postive |
| TRPC6 | MACORIS | 0.470835526 | 3.30E-11 | postive |
| TRPV6 | LINC00987 | 0.609727831 | 1.67E-19 | postive |
| TRPA1 | HOXA-AS3 | 0.712989561 | 6.15E-29 | postive |
| TRPM5 | AC138356.1 | 0.536543784 | 1.17E-14 | postive |
| MCOLN3 | AC138356.1 | 0.556540288 | 7.26E-16 | postive |
| MCOLN2 | AL023653.1 | 0.812107043 | 4.92E-43 | postive |
| TRPC1 | ZBED3-AS1 | 0.413387054 | 9.75E-09 | postive |
| TRPM3 | ZBED3-AS1 | 0.413241052 | 9.88E-09 | postive |
| MCOLN1 | ZBED3-AS1 | 0.640902307 | 5.68E-22 | postive |
| TRPV4 | AC068580.2 | 0.420988591 | 4.88E-09 | postive |
| TRPC1 | ST3GAL5-AS1 | 0.454053381 | 1.94E-10 | postive |
| TRPC6 | ST3GAL5-AS1 | 0.456724848 | 1.48E-10 | postive |
| MCOLN1 | ST3GAL5-AS1 | 0.444036393 | 5.35E-10 | postive |
| PKD2 | ST3GAL5-AS1 | 0.406517884 | 1.80E-08 | postive |
| TRPM4 | AL606489.1 | 0.528788332 | 3.27E-14 | postive |
| TRPV6 | LINC02212 | 0.475227552 | 2.05E-11 | postive |
| MCOLN2 | AC243829.1 | 0.574904261 | 4.78E-17 | postive |
| TRPA1 | AC069549.1 | 0.610050096 | 1.58E-19 | postive |
| TRPM5 | AL672291.1 | 0.92656704 | 1.18E-76 | postive |
| MCOLN3 | AL672291.1 | 0.81958663 | 1.96E-44 | postive |
| MCOLN2 | LINC02158 | 0.405151817 | 2.03E-08 | postive |
| PKD1 | ZNF710-AS1 | 0.631112144 | 3.63E-21 | postive |
| TRPC6 | LINC00840 | 0.428010855 | 2.53E-09 | postive |
| MCOLN2 | AC079793.1 | 0.737137815 | 8.92E-32 | postive |
| TRPC2 | AC004921.1 | 0.557631117 | 6.21E-16 | postive |
| TRPC6 | AC004921.1 | 0.465332794 | 5.97E-11 | postive |
| TRPM2 | AC004921.1 | 0.5174524 | 1.41E-13 | postive |
| PKD2 | AC004921.1 | 0.415958039 | 7.73E-09 | postive |
| PKD2L1 | AC004921.1 | 0.480462658 | 1.14E-11 | postive |
| TRPV2 | AC004921.1 | 0.412551714 | 1.05E-08 | postive |
| TRPC3 | LINC02126 | 0.457820125 | 1.32E-10 | postive |
| PKD2 | LINC02126 | 0.443354589 | 5.73E-10 | postive |
| MCOLN2 | AP005131.2 | 0.526862501 | 4.21E-14 | postive |
| TRPM4 | SYNPR-AS1 | 0.465242646 | 6.03E-11 | postive |
| TRPC7 | AP000894.4 | 0.562532746 | 3.04E-16 | postive |
| TRPM5 | AP000894.4 | 0.525994052 | 4.71E-14 | postive |
| MCOLN3 | AP000894.4 | 0.65092883 | 7.91E-23 | postive |
| PKD1 | AP000894.4 | 0.455566758 | 1.66E-10 | postive |
| TRPC3 | AC006033.2 | 0.40704645 | 1.72E-08 | postive |
| TRPM2 | AC006033.2 | 0.513133751 | 2.42E-13 | postive |
| TRPM7 | AC006033.2 | 0.508577169 | 4.24E-13 | postive |
| MCOLN2 | AC006033.2 | 0.430714166 | 1.96E-09 | postive |
| PKD2 | AC006033.2 | 0.582111523 | 1.57E-17 | postive |
| TRPV2 | AC006033.2 | 0.461958007 | 8.54E-11 | postive |
| TRPC1 | WT1-AS | 0.597391327 | 1.34E-18 | postive |
| TRPC3 | WT1-AS | 0.510666819 | 3.28E-13 | postive |
| PKD2 | WT1-AS | 0.591149495 | 3.72E-18 | postive |
| MCOLN2 | ZBTB20-AS1 | 0.571192943 | 8.40E-17 | postive |
| TRPM2 | AC002091.2 | 0.620583261 | 2.48E-20 | postive |
| TRPM7 | AC002091.2 | 0.440320384 | 7.73E-10 | postive |
| MCOLN2 | AC002091.2 | 0.590950208 | 3.84E-18 | postive |
| PKD2 | AC002091.2 | 0.452300171 | 2.33E-10 | postive |
| PKD2L1 | AC002091.2 | 0.443800798 | 5.48E-10 | postive |
| TRPV1 | MRPS9-AS1 | 0.510472072 | 3.36E-13 | postive |
| TRPC2 | AC063977.6 | 0.535850306 | 1.28E-14 | postive |
| PKD2L1 | AC063977.6 | 0.503965732 | 7.43E-13 | postive |
| TRPC1 | AC134312.1 | 0.430927337 | 1.92E-09 | postive |
| PKD2 | AC134312.1 | 0.456550133 | 1.50E-10 | postive |
| TRPV1 | AC020612.3 | 0.464761308 | 6.35E-11 | postive |
| TRPC2 | AC009690.2 | 0.459125041 | 1.15E-10 | postive |
| MCOLN2 | AC009690.2 | 0.466925886 | 5.04E-11 | postive |
| TRPV1 | AC009690.2 | 0.470281491 | 3.51E-11 | postive |
| TRPC2 | AC007336.2 | 0.577385731 | 3.27E-17 | postive |
| PKD2L1 | AC007336.2 | 0.711887427 | 8.15E-29 | postive |
| TRPM2 | C22orf34 | 0.400151167 | 3.13E-08 | postive |
| MCOLN2 | C22orf34 | 0.616689362 | 4.97E-20 | postive |
| MCOLN2 | AC010894.4 | 0.779056072 | 1.58E-37 | postive |
| TRPV6 | PRKAR1B-AS2 | 0.659951885 | 1.26E-23 | postive |
| TRPC3 | ST6GAL2-IT1 | 0.433459078 | 1.51E-09 | postive |
| PKD2 | AC116351.1 | 0.456686343 | 1.48E-10 | postive |
| TRPM5 | AF131215.7 | 0.482001421 | 9.62E-12 | postive |
| MCOLN3 | AF131215.7 | 0.478034862 | 1.50E-11 | postive |
| TRPM2 | AC008759.3 | 0.401167314 | 2.87E-08 | postive |
| TRPM4 | TRIM31-AS1 | 0.440933394 | 7.28E-10 | postive |
| TRPA1 | AC093904.2 | 0.454868195 | 1.79E-10 | postive |
| TRPM4 | AC093904.2 | 0.406753076 | 1.76E-08 | postive |
| TRPC6 | AL672032.1 | 0.422904667 | 4.08E-09 | postive |
| PKD2 | AL672032.1 | 0.491815523 | 3.14E-12 | postive |
| TRPC1 | AL158163.2 | 0.401197938 | 2.86E-08 | postive |
| TRPC7 | AL158163.2 | 0.497925383 | 1.53E-12 | postive |
| MCOLN1 | AL158163.2 | 0.443016341 | 5.92E-10 | postive |
| MCOLN3 | AL158163.2 | 0.457443539 | 1.37E-10 | postive |
| PKD1 | AL158163.2 | 0.559919391 | 4.46E-16 | postive |
| MCOLN2 | Z84723.1 | 0.764392166 | 2.22E-35 | postive |
| TRPM2 | AC079209.1 | 0.428432062 | 2.43E-09 | postive |
| MCOLN2 | AC079209.1 | 0.5001656 | 1.17E-12 | postive |
| PKD2L1 | AC079209.1 | 0.416964608 | 7.05E-09 | postive |
| TRPA1 | AC093904.3 | 0.408775093 | 1.47E-08 | postive |
| TRPV3 | AC004264.1 | 0.46219135 | 8.33E-11 | postive |
| TRPC3 | LCT-AS1 | 0.470274291 | 3.51E-11 | postive |
| TRPC1 | LINC01410 | 0.411887754 | 1.12E-08 | postive |
| MCOLN1 | LINC01410 | 0.475006688 | 2.10E-11 | postive |
| MCOLN2 | LINC01410 | 0.413248609 | 9.87E-09 | postive |
| MCOLN2 | AC011377.1 | 0.450144215 | 2.90E-10 | postive |
| TRPM2 | AC034199.1 | 0.404028025 | 2.24E-08 | postive |
| TRPC2 | AP002954.1 | 0.547581665 | 2.58E-15 | postive |
| TRPM2 | AP002954.1 | 0.468524838 | 4.24E-11 | postive |
| PKD2L1 | AP002954.1 | 0.518866991 | 1.18E-13 | postive |
| TRPV2 | AP002954.1 | 0.468088531 | 4.45E-11 | postive |
| MCOLN2 | AC008680.1 | 0.530651079 | 2.56E-14 | postive |
| TRPC1 | AC037198.2 | 0.57855862 | 2.72E-17 | postive |
| TRPC6 | AC037198.2 | 0.448035465 | 3.59E-10 | postive |
| PKD2 | AC037198.2 | 0.476450269 | 1.79E-11 | postive |
| TRPM5 | AC027228.2 | 0.673395235 | 7.21E-25 | postive |
| MCOLN3 | AC027228.2 | 0.48992277 | 3.90E-12 | postive |
| TRPV4 | AC078923.1 | 0.498153461 | 1.49E-12 | postive |
| TRPC1 | NEXN-AS1 | 0.595028718 | 1.98E-18 | postive |
| PKD1 | NEXN-AS1 | 0.56072754 | 3.96E-16 | postive |
| TRPM2 | LINC02084 | 0.464967093 | 6.21E-11 | postive |
| MCOLN2 | LINC02084 | 0.739169404 | 4.98E-32 | postive |
| TRPV2 | AC019254.1 | 0.462505883 | 8.06E-11 | postive |
| TRPM2 | LINC01684 | 0.465964145 | 5.58E-11 | postive |
| TRPM7 | LINC01684 | 0.475324749 | 2.02E-11 | postive |
| MCOLN2 | LINC01684 | 0.711282084 | 9.51E-29 | postive |
| PKD2 | LINC01684 | 0.466656781 | 5.18E-11 | postive |
| TRPA1 | AL117382.2 | 0.411913656 | 1.11E-08 | postive |
| TRPC1 | LINC02705 | 0.403200277 | 2.40E-08 | postive |
| TRPM7 | LINC02705 | 0.426080859 | 3.04E-09 | postive |
| PKD2 | LINC02705 | 0.463468265 | 7.28E-11 | postive |
| TRPC6 | AC069209.1 | 0.417953548 | 6.44E-09 | postive |
| TRPC1 | ACTA2-AS1 | 0.464732849 | 6.37E-11 | postive |
| TRPC3 | ACTA2-AS1 | 0.490471416 | 3.66E-12 | postive |
| PKD2 | ACTA2-AS1 | 0.470445874 | 3.45E-11 | postive |
| MCOLN2 | AP001636.3 | 0.483116638 | 8.49E-12 | postive |
| MCOLN2 | AC068481.1 | 0.422340283 | 4.30E-09 | postive |
| TRPC7 | LINC02251 | 0.433436387 | 1.51E-09 | postive |
| TRPM3 | LINC02251 | 0.464146559 | 6.78E-11 | postive |
| MCOLN1 | LINC02251 | 0.409407073 | 1.39E-08 | postive |
| MCOLN2 | LINC02251 | 0.431051577 | 1.90E-09 | postive |
| TRPM2 | AP000812.1 | 0.42067176 | 5.02E-09 | postive |
| MCOLN2 | LINC00092 | 0.638733407 | 8.62E-22 | postive |
| TRPM4 | AP005233.2 | 0.400600934 | 3.01E-08 | postive |
| TRPC7 | AF250324.1 | 0.443196826 | 5.82E-10 | postive |
| TRPM5 | AC103855.3 | 0.973081873 | 4.05E-114 | postive |
| MCOLN3 | AC103855.3 | 0.787668795 | 7.23E-39 | postive |
| TRPV6 | AC005381.1 | 0.42418465 | 3.62E-09 | postive |
| TRPA1 | BCAR3-AS1 | 0.498375913 | 1.45E-12 | postive |
| MCOLN2 | AC005332.2 | 0.707536465 | 2.45E-28 | postive |
| MCOLN2 | LINC01857 | 0.813075094 | 3.27E-43 | postive |
| TRPC2 | AC010904.2 | 0.446102714 | 4.36E-10 | postive |
| TRPA1 | CASC9 | 0.453068968 | 2.15E-10 | postive |
| TRPC1 | LINC02773 | 0.448965169 | 3.27E-10 | postive |
| TRPC6 | LINC02773 | 0.523067453 | 6.87E-14 | postive |
| TRPM2 | LINC02773 | 0.432473091 | 1.66E-09 | postive |
| TRPM7 | LINC02773 | 0.438564591 | 9.18E-10 | postive |
| MCOLN2 | LINC02773 | 0.444406004 | 5.16E-10 | postive |
| PKD2 | LINC02773 | 0.556842484 | 6.95E-16 | postive |
| TRPM5 | AL359233.1 | 0.419465339 | 5.61E-09 | postive |
| MCOLN3 | AL359233.1 | 0.440834583 | 7.35E-10 | postive |
| PKD1 | AL359233.1 | 0.514524771 | 2.03E-13 | postive |
| TRPC4 | AP000802.1 | 0.449751213 | 3.02E-10 | postive |
| TRPC7 | AP000802.1 | 0.733836159 | 2.27E-31 | postive |
| TRPM3 | AP000802.1 | 0.446887084 | 4.03E-10 | postive |
| TRPM5 | AP000802.1 | 0.77811824 | 2.19E-37 | postive |
| MCOLN3 | AP000802.1 | 0.884190134 | 4.49E-60 | postive |
| PKD1 | AP000802.1 | 0.470392073 | 3.47E-11 | postive |
| TRPC1 | AC090559.1 | 0.478766361 | 1.38E-11 | postive |
| TRPC6 | AC090559.1 | 0.566771328 | 1.63E-16 | postive |
| TRPM2 | AC090559.1 | 0.533231638 | 1.82E-14 | postive |
| TRPM7 | AC090559.1 | 0.603646698 | 4.73E-19 | postive |
| MCOLN2 | AC090559.1 | 0.456157018 | 1.57E-10 | postive |
| PKD2 | AC090559.1 | 0.69751799 | 2.87E-27 | postive |
| TRPV1 | AC005104.1 | 0.499311679 | 1.30E-12 | postive |
| TRPC1 | DIRC3-AS1 | 0.453911199 | 1.97E-10 | postive |
| MCOLN2 | AC084876.1 | 0.594602394 | 2.12E-18 | postive |
| MCOLN1 | AL354809.1 | 0.412939007 | 1.02E-08 | postive |
| PKD1 | AL354809.1 | 0.476177185 | 1.84E-11 | postive |
| TRPV1 | AL354809.1 | 0.418430073 | 6.17E-09 | postive |
| TRPC6 | MIR223HG | 0.403082225 | 2.43E-08 | postive |
| MCOLN2 | MIR223HG | 0.483926623 | 7.75E-12 | postive |
| MCOLN2 | AC023510.1 | 0.627607 | 6.95E-21 | postive |
| MCOLN3 | AC037487.1 | 0.415159841 | 8.31E-09 | postive |
| TRPV1 | AC037487.1 | 0.425976154 | 3.07E-09 | postive |
| PKD1 | AC002480.1 | 0.435578274 | 1.23E-09 | postive |
| TRPC2 | AC138207.4 | 0.522309341 | 7.58E-14 | postive |
| TRPM2 | AC138207.4 | 0.434182744 | 1.40E-09 | postive |
| PKD2 | AC138207.4 | 0.426946949 | 2.80E-09 | postive |
| TRPC7 | AC005089.1 | 0.547724838 | 2.53E-15 | postive |
| TRPM3 | AC005089.1 | 0.574644468 | 4.97E-17 | postive |
| MCOLN1 | AC005089.1 | 0.518322103 | 1.26E-13 | postive |
| TRPC7 | AC096733.2 | 0.68754434 | 3.01E-26 | postive |
| TRPM3 | AC096733.2 | 0.676477389 | 3.66E-25 | postive |
| MCOLN1 | AC096733.2 | 0.475542199 | 1.98E-11 | postive |
| MCOLN3 | AC096733.2 | 0.443292783 | 5.76E-10 | postive |
| PKD1 | AC096733.2 | 0.50368019 | 7.70E-13 | postive |
| MCOLN1 | PAN3-AS1 | 0.430997114 | 1.91E-09 | postive |
| MCOLN3 | PAN3-AS1 | 0.505746752 | 5.99E-13 | postive |
| PKD1 | PAN3-AS1 | 0.460099602 | 1.04E-10 | postive |
| TRPV1 | PAN3-AS1 | 0.400462159 | 3.05E-08 | postive |
| TRPC2 | LINC02785 | 0.541653878 | 5.85E-15 | postive |
| PKD2L1 | LINC02785 | 0.486299649 | 5.91E-12 | postive |
| TRPV2 | LINC02785 | 0.436689747 | 1.10E-09 | postive |
| TRPC6 | LINC01150 | 0.540236966 | 7.09E-15 | postive |
| TRPM7 | LINC01150 | 0.453133422 | 2.14E-10 | postive |
| PKD2 | LINC01150 | 0.536574679 | 1.16E-14 | postive |
| TRPV1 | AL807752.4 | 0.40839411 | 1.52E-08 | postive |
| TRPM7 | TGFB3-AS1 | 0.425103648 | 3.33E-09 | postive |
| PKD2 | TGFB3-AS1 | 0.454274782 | 1.90E-10 | postive |
| TRPC7 | AC007786.1 | 0.414182644 | 9.08E-09 | postive |
| MCOLN3 | AC007786.1 | 0.401734839 | 2.73E-08 | postive |
| PKD1 | AC007786.1 | 0.423196858 | 3.97E-09 | postive |
| MCOLN1 | AL359532.1 | 0.432465708 | 1.66E-09 | postive |
| PKD1 | AL359532.1 | 0.409295744 | 1.41E-08 | postive |
| MCOLN2 | AC027097.1 | 0.815510226 | 1.16E-43 | postive |
| TRPM5 | AC079035.1 | 0.971652085 | 3.61E-112 | postive |
| MCOLN3 | AC079035.1 | 0.785095053 | 1.85E-38 | postive |
| TRPM5 | AC104117.5 | 0.52962733 | 2.93E-14 | postive |
| MCOLN3 | AC104117.5 | 0.664636124 | 4.73E-24 | postive |
| MCOLN2 | AC090948.3 | 0.820667146 | 1.22E-44 | postive |
| TRPM5 | FAM27E3 | 0.443203675 | 5.82E-10 | postive |
| MCOLN3 | FAM27E3 | 0.505676758 | 6.04E-13 | postive |
| TRPM7 | AL137003.1 | 0.450684806 | 2.74E-10 | postive |
| MCOLN2 | LINC01771 | 0.497828872 | 1.55E-12 | postive |
| TRPC1 | ZEB2-AS1 | 0.403073968 | 2.43E-08 | postive |
| TRPC2 | ZEB2-AS1 | 0.505724425 | 6.01E-13 | postive |
| TRPC6 | ZEB2-AS1 | 0.479827549 | 1.23E-11 | postive |
| TRPM2 | ZEB2-AS1 | 0.501406119 | 1.01E-12 | postive |
| MCOLN2 | ZEB2-AS1 | 0.533448143 | 1.77E-14 | postive |
| PKD2 | ZEB2-AS1 | 0.429738951 | 2.15E-09 | postive |
| MCOLN1 | AC100810.1 | 0.484160936 | 7.54E-12 | postive |
| PKD1 | AC100810.1 | 0.413627417 | 9.54E-09 | postive |
| MCOLN2 | LINC02361 | 0.570020768 | 1.00E-16 | postive |
| TRPA1 | LINC02747 | 0.524781923 | 5.51E-14 | postive |
| TRPV2 | AC093484.1 | 0.451934236 | 2.42E-10 | postive |
| TRPV6 | AC118754.1 | 0.414678818 | 8.68E-09 | postive |
| TRPC1 | DNM3OS | 0.607723022 | 2.36E-19 | postive |
| TRPC3 | DNM3OS | 0.543079088 | 4.81E-15 | postive |
| TRPC6 | DNM3OS | 0.461586033 | 8.88E-11 | postive |
| TRPM7 | DNM3OS | 0.408088205 | 1.56E-08 | postive |
| PKD2 | DNM3OS | 0.651161595 | 7.55E-23 | postive |
| TRPC7 | AC079385.3 | 0.408158002 | 1.55E-08 | postive |
| PKD1 | AC079385.3 | 0.516228442 | 1.64E-13 | postive |
| MCOLN2 | PCED1B-AS1 | 0.871131945 | 2.99E-56 | postive |
| TRPC2 | C9orf139 | 0.44505943 | 4.84E-10 | postive |
| TRPM2 | C9orf139 | 0.40230639 | 2.60E-08 | postive |
| MCOLN1 | C9orf139 | 0.479240866 | 1.31E-11 | postive |
| MCOLN2 | C9orf139 | 0.576981816 | 3.48E-17 | postive |
| TRPM2 | AC145098.1 | 0.440717413 | 7.44E-10 | postive |
| MCOLN2 | AC145098.1 | 0.758438045 | 1.49E-34 | postive |
| TRPM2 | AC021188.1 | 0.400203825 | 3.12E-08 | postive |
| MCOLN2 | AC021188.1 | 0.753207155 | 7.63E-34 | postive |
| TRPC7 | TRIM52-AS1 | 0.629642798 | 4.77E-21 | postive |
| TRPM3 | TRIM52-AS1 | 0.48412847 | 7.57E-12 | postive |
| MCOLN1 | TRIM52-AS1 | 0.533175487 | 1.83E-14 | postive |
| MCOLN3 | TRIM52-AS1 | 0.554609132 | 9.58E-16 | postive |
| PKD1 | TRIM52-AS1 | 0.506033045 | 5.79E-13 | postive |
| TRPA1 | LINC02562 | 0.406695152 | 1.77E-08 | postive |
| MCOLN2 | Z82188.2 | 0.495693665 | 1.99E-12 | postive |
| TRPV4 | C20orf197 | 0.422123691 | 4.39E-09 | postive |
| TRPM5 | LINC02593 | 0.964306654 | 1.68E-103 | postive |
| MCOLN3 | LINC02593 | 0.815323683 | 1.25E-43 | postive |
| TRPM5 | C2orf91 | 0.696442504 | 3.71E-27 | postive |
| MCOLN3 | C2orf91 | 0.578318288 | 2.83E-17 | postive |
| TRPA1 | AC093904.4 | 0.588868173 | 5.37E-18 | postive |
| TRPM4 | AC012317.1 | 0.455655911 | 1.65E-10 | postive |
| TRPM5 | AC020663.2 | 0.461613082 | 8.86E-11 | postive |
| TRPC2 | AC008750.1 | 0.484043665 | 7.64E-12 | postive |
| TRPM2 | AC008750.1 | 0.502918421 | 8.44E-13 | postive |
| MCOLN2 | AC008750.1 | 0.678926904 | 2.13E-25 | postive |
| TRPM2 | LINC02285 | 0.49195473 | 3.08E-12 | postive |
| MCOLN2 | LINC02285 | 0.759150195 | 1.19E-34 | postive |
| MCOLN2 | AC015795.1 | 0.478454199 | 1.43E-11 | postive |
| TRPC7 | AC087501.4 | 0.416844968 | 7.13E-09 | postive |
| MCOLN1 | AC087501.4 | 0.483243667 | 8.37E-12 | postive |
| MCOLN3 | AC087501.4 | 0.43038688 | 2.02E-09 | postive |
| PKD1 | AC087501.4 | 0.402010442 | 2.66E-08 | postive |
| TRPV1 | GLYCTK-AS1 | 0.458211419 | 1.26E-10 | postive |
| TRPV6 | GLYCTK-AS1 | 0.45714199 | 1.41E-10 | postive |
| TRPC1 | AP001596.1 | 0.423418149 | 3.89E-09 | postive |
| TRPC6 | AP001596.1 | 0.567713392 | 1.42E-16 | postive |
| TRPC6 | AC022150.3 | 0.42126535 | 4.75E-09 | postive |
| PKD2 | AC022150.3 | 0.475205339 | 2.05E-11 | postive |
| PKD1 | MEG9 | 0.532581126 | 1.98E-14 | postive |
| TRPC1 | LINC02256 | 0.5717858 | 7.68E-17 | postive |
| TRPM7 | LINC02256 | 0.409088867 | 1.43E-08 | postive |
| MCOLN1 | LINC02256 | 0.450499068 | 2.80E-10 | postive |
| PKD1 | LINC02256 | 0.430551625 | 1.99E-09 | postive |
| PKD2 | LINC02256 | 0.488872685 | 4.40E-12 | postive |
| TRPC1 | AF111169.3 | 0.415977291 | 7.71E-09 | postive |
| TRPC7 | AF111169.3 | 0.466306997 | 5.38E-11 | postive |
| TRPM5 | AF111169.3 | 0.658204461 | 1.81E-23 | postive |
| TRPM6 | AF111169.3 | 0.406213901 | 1.85E-08 | postive |
| MCOLN1 | AF111169.3 | 0.499923329 | 1.21E-12 | postive |
| MCOLN3 | AF111169.3 | 0.719153078 | 1.24E-29 | postive |
| PKD1 | AF111169.3 | 0.535232967 | 1.39E-14 | postive |
| TRPV1 | AF111169.3 | 0.455089401 | 1.75E-10 | postive |
| TRPC7 | HEIH | 0.652419214 | 5.87E-23 | postive |
| TRPM3 | HEIH | 0.41908222 | 5.81E-09 | postive |
| MCOLN1 | HEIH | 0.530395344 | 2.65E-14 | postive |
| MCOLN3 | HEIH | 0.512950912 | 2.47E-13 | postive |
| PKD1 | HEIH | 0.497475029 | 1.62E-12 | postive |
| MCOLN2 | AL117336.1 | 0.465535803 | 5.84E-11 | postive |
| TRPM2 | TRG-AS1 | 0.46317922 | 7.51E-11 | postive |
| TRPM7 | TRG-AS1 | 0.415402653 | 8.13E-09 | postive |
| MCOLN2 | TRG-AS1 | 0.877402166 | 4.95E-58 | postive |
| TRPM5 | AC245140.1 | 0.402443081 | 2.57E-08 | postive |
| MCOLN3 | AC245140.1 | 0.501047551 | 1.06E-12 | postive |
| TRPV3 | LIF-AS1 | 0.410301297 | 1.29E-08 | postive |
| MCOLN2 | AC087045.2 | 0.599819857 | 8.98E-19 | postive |
| TRPV1 | AC138028.4 | 0.514241111 | 2.10E-13 | postive |
| TRPC2 | AC011899.2 | 0.590564707 | 4.09E-18 | postive |
| TRPV2 | AC011899.2 | 0.432121334 | 1.71E-09 | postive |
| TRPC2 | ADPGK-AS1 | 0.420792112 | 4.97E-09 | postive |
| TRPM2 | ADPGK-AS1 | 0.487440393 | 5.19E-12 | postive |
| MCOLN2 | ADPGK-AS1 | 0.680720035 | 1.42E-25 | postive |
| TRPV2 | ADPGK-AS1 | 0.437525446 | 1.02E-09 | postive |
| TRPC4 | TRPC7-AS1 | 0.531911478 | 2.17E-14 | postive |
| TRPC7 | TRPC7-AS1 | 0.750331525 | 1.84E-33 | postive |
| MCOLN3 | TRPC7-AS1 | 0.547269728 | 2.69E-15 | postive |
| PKD1 | TRPC7-AS1 | 0.424998996 | 3.36E-09 | postive |
| MCOLN2 | Z97192.3 | 0.732722969 | 3.11E-31 | postive |
| TRPM5 | AC009812.4 | 0.441081128 | 7.17E-10 | postive |
| MCOLN3 | AC009812.4 | 0.454432802 | 1.87E-10 | postive |
| MCOLN2 | GHRLOS | 0.438516187 | 9.23E-10 | postive |
| TRPM2 | AL133371.2 | 0.480174029 | 1.18E-11 | postive |
| MCOLN2 | AL133371.2 | 0.636230662 | 1.39E-21 | postive |
| TRPM4 | LINC02323 | 0.425346414 | 3.25E-09 | postive |
| TRPC4 | AL356215.1 | 0.47018592 | 3.55E-11 | postive |
| TRPM2 | HDAC2-AS2 | 0.484865128 | 6.96E-12 | postive |
| TRPC1 | AC005332.4 | 0.443745156 | 5.51E-10 | postive |
| MCOLN2 | AC005332.4 | 0.657742867 | 1.99E-23 | postive |
| TRPA1 | SLCO4A1-AS1 | 0.505317791 | 6.31E-13 | postive |
| TRPC1 | ZNF582-AS1 | 0.529833664 | 2.85E-14 | postive |
| TRPC6 | ZNF582-AS1 | 0.518164684 | 1.28E-13 | postive |
| MCOLN1 | ZNF582-AS1 | 0.531318343 | 2.35E-14 | postive |
| PKD1 | ZNF582-AS1 | 0.40825742 | 1.54E-08 | postive |
| PKD2 | ZNF582-AS1 | 0.403133336 | 2.42E-08 | postive |
| TRPM4 | DCST1-AS1 | 0.416263221 | 7.52E-09 | postive |
| TRPC6 | AC074135.1 | 0.457667278 | 1.34E-10 | postive |
| PKD2 | AC074135.1 | 0.438035969 | 9.67E-10 | postive |
| MCOLN2 | AC010175.1 | 0.658937002 | 1.55E-23 | postive |
| TRPC2 | AC025048.4 | 0.414244179 | 9.02E-09 | postive |
| TRPV1 | AL121894.2 | 0.470497609 | 3.43E-11 | postive |
| TRPV1 | AC129510.1 | 0.66226729 | 7.77E-24 | postive |
| PKD1 | MIAT | 0.420554924 | 5.07E-09 | postive |
| PKD1 | AL355601.1 | 0.441567689 | 6.84E-10 | postive |
| MCOLN2 | LINC01754 | 0.669547446 | 1.66E-24 | postive |
| TRPM2 | PIK3CD-AS1 | 0.404480137 | 2.15E-08 | postive |
| MCOLN2 | PIK3CD-AS1 | 0.811162824 | 7.31E-43 | postive |
| TRPM5 | Z97832.2 | 0.463340078 | 7.38E-11 | postive |
| MCOLN3 | Z97832.2 | 0.546862876 | 2.85E-15 | postive |
| PKD1 | Z97832.2 | 0.577451212 | 3.23E-17 | postive |
| TRPV1 | Z97832.2 | 0.5855579 | 9.11E-18 | postive |
| TRPM4 | AL118505.1 | 0.414270977 | 9.00E-09 | postive |
| TRPM4 | AP001107.9 | 0.403179707 | 2.41E-08 | postive |
| TRPC4 | ZNF236-DT | 0.429808607 | 2.13E-09 | postive |
| MCOLN1 | ZNF236-DT | 0.583090128 | 1.34E-17 | postive |
| PKD1 | ZNF236-DT | 0.499599536 | 1.26E-12 | postive |
| TRPM8 | AC034213.1 | 0.561302578 | 3.64E-16 | postive |
| PKD1 | AC005920.3 | 0.414106362 | 9.14E-09 | postive |
| MCOLN2 | AOAH-IT1 | 0.612612787 | 1.02E-19 | postive |
| TRPM2 | AC022706.1 | 0.463526218 | 7.24E-11 | postive |
| MCOLN2 | AC022706.1 | 0.59711551 | 1.41E-18 | postive |
| TRPV2 | AC022706.1 | 0.469119529 | 3.98E-11 | postive |
| MCOLN2 | GTSCR1 | 0.678945408 | 2.12E-25 | postive |
| TRPC1 | RBMS3-AS3 | 0.412969836 | 1.01E-08 | postive |
| TRPC6 | RBMS3-AS3 | 0.435053872 | 1.29E-09 | postive |
| PKD2 | RBMS3-AS3 | 0.417353932 | 6.81E-09 | postive |
| PKD1 | LINC01954 | 0.406018302 | 1.88E-08 | postive |
| TRPM5 | LINC02600 | 0.971100462 | 1.92E-111 | postive |
| MCOLN3 | LINC02600 | 0.814852449 | 1.53E-43 | postive |
| TRPM5 | INTS6L-AS1 | 0.495017626 | 2.16E-12 | postive |
| MCOLN3 | INTS6L-AS1 | 0.450758941 | 2.72E-10 | postive |
| TRPC7 | LINC00847 | 0.429135607 | 2.28E-09 | postive |
| MCOLN1 | LINC00847 | 0.453666993 | 2.02E-10 | postive |
| TRPM5 | AC078864.1 | 0.666115309 | 3.46E-24 | postive |
| MCOLN3 | AC078864.1 | 0.582176804 | 1.55E-17 | postive |
| PKD1 | AC078864.1 | 0.526235164 | 4.57E-14 | postive |
| TRPC2 | U62317.1 | 0.474996876 | 2.10E-11 | postive |
| TRPV2 | AC023301.1 | 0.403166941 | 2.41E-08 | postive |
| TRPM4 | LINC01814 | 0.499244934 | 1.31E-12 | postive |
| MCOLN1 | AC002059.1 | 0.465095712 | 6.12E-11 | postive |
| MCOLN2 | AC002059.1 | 0.401314422 | 2.83E-08 | postive |
| TRPA1 | KDM7A-DT | 0.748423111 | 3.27E-33 | postive |
| TRPC1 | STARD4-AS1 | 0.60657546 | 2.88E-19 | postive |
| TRPC3 | STARD4-AS1 | 0.488423098 | 4.64E-12 | postive |
| TRPC6 | STARD4-AS1 | 0.400828165 | 2.95E-08 | postive |
| TRPM7 | STARD4-AS1 | 0.589884787 | 4.56E-18 | postive |
| PKD1 | STARD4-AS1 | 0.444984339 | 4.87E-10 | postive |
| PKD2 | STARD4-AS1 | 0.638713975 | 8.65E-22 | postive |
| TRPC2 | AC078850.1 | 0.408087884 | 1.56E-08 | postive |
| TRPC1 | AC009831.1 | 0.510812359 | 3.22E-13 | postive |
| PKD2 | AC009831.1 | 0.443974823 | 5.39E-10 | postive |
| TRPM8 | LINC00973 | 0.427994893 | 2.54E-09 | postive |
| MCOLN2 | AC007728.2 | 0.827068356 | 6.72E-46 | postive |
| TRPC2 | COL4A2-AS2 | 0.437535682 | 1.02E-09 | postive |
| PKD2L1 | COL4A2-AS2 | 0.622197367 | 1.86E-20 | postive |
| TRPM4 | MIR194-2HG | 0.506658262 | 5.36E-13 | postive |
| TRPM4 | LINC02041 | 0.525540981 | 5.00E-14 | postive |
| TRPA1 | AC009065.5 | 0.471191244 | 3.18E-11 | postive |
| TRPC1 | AC009065.5 | -0.409389068 | 1.39E-08 | negative |
| TRPM4 | AC009065.5 | 0.531399821 | 2.32E-14 | postive |
| PKD2 | LINC02613 | 0.44803927 | 3.59E-10 | postive |
| MCOLN2 | AC012645.3 | 0.840544631 | 1.00E-48 | postive |
| MCOLN2 | Z97192.2 | 0.481214756 | 1.05E-11 | postive |
| TRPM4 | AC104964.1 | 0.401556399 | 2.77E-08 | postive |
| TRPC1 | AL139260.1 | 0.454330263 | 1.89E-10 | postive |
| TRPC6 | AL139260.1 | 0.428389204 | 2.44E-09 | postive |
| MCOLN1 | AL139260.1 | 0.469749288 | 3.72E-11 | postive |
| MCOLN2 | AC055822.1 | 0.660396251 | 1.15E-23 | postive |
| TRPA1 | AC109446.3 | 0.492945382 | 2.75E-12 | postive |
| MCOLN2 | AC109446.3 | 0.564845069 | 2.17E-16 | postive |
| TRPC2 | FAM225A | 0.478401572 | 1.44E-11 | postive |
| TRPV2 | FAM225A | 0.471079701 | 3.22E-11 | postive |
| TRPM4 | AL022322.1 | 0.504392421 | 7.06E-13 | postive |
| TRPV1 | AL022322.1 | 0.44085879 | 7.33E-10 | postive |
| TRPM5 | AC013400.1 | 0.574576606 | 5.02E-17 | postive |
| MCOLN3 | AC013400.1 | 0.617940327 | 3.98E-20 | postive |
| TRPC7 | GRM3-AS1 | 0.426619012 | 2.89E-09 | postive |
| TRPC1 | AC008840.1 | 0.48913333 | 4.27E-12 | postive |
| TRPC3 | AC008840.1 | 0.405039649 | 2.05E-08 | postive |
| TRPC6 | AC008840.1 | 0.402172302 | 2.63E-08 | postive |
| PKD2 | AC008840.1 | 0.558670565 | 5.34E-16 | postive |
| TRPC4 | AC068987.3 | 0.407729425 | 1.62E-08 | postive |
| MCOLN3 | AC068987.3 | 0.456138165 | 1.57E-10 | postive |
| TRPA1 | AL024508.1 | 0.7225192 | 5.06E-30 | postive |
| MCOLN1 | AC016590.1 | 0.440779222 | 7.39E-10 | postive |
| TRPC2 | AC060766.4 | 0.418018832 | 6.41E-09 | postive |
| TRPC1 | WAKMAR2 | 0.435186825 | 1.28E-09 | postive |
| TRPC6 | WAKMAR2 | 0.495346233 | 2.08E-12 | postive |
| TRPM2 | WAKMAR2 | 0.453981066 | 1.96E-10 | postive |
| TRPM7 | WAKMAR2 | 0.437968073 | 9.73E-10 | postive |
| MCOLN2 | WAKMAR2 | 0.766092302 | 1.28E-35 | postive |
| PKD2 | WAKMAR2 | 0.438874643 | 8.91E-10 | postive |
| TRPC6 | AC104984.4 | 0.457362516 | 1.38E-10 | postive |
| TRPV4 | AL121772.1 | 0.432562301 | 1.64E-09 | postive |
| TRPC2 | AL137186.2 | 0.459124103 | 1.15E-10 | postive |
| MCOLN2 | LINC01160 | 0.455830026 | 1.62E-10 | postive |
| PKD1 | LINC01160 | 0.445232438 | 4.75E-10 | postive |
| TRPV6 | AC067817.2 | 0.531183537 | 2.39E-14 | postive |
| TRPA1 | LINC01124 | 0.64144691 | 5.11E-22 | postive |
| TRPC2 | MMP2-AS1 | 0.40416458 | 2.21E-08 | postive |
| PKD2L1 | MMP2-AS1 | 0.41727843 | 6.85E-09 | postive |
| TRPC2 | AL137026.1 | 0.403046424 | 2.44E-08 | postive |
| TRPM4 | OVOL1-AS1 | 0.414061648 | 9.17E-09 | postive |
| TRPC1 | TRAF3IP2-AS1 | 0.611487148 | 1.24E-19 | postive |
| TRPC4 | TRAF3IP2-AS1 | 0.520230075 | 9.88E-14 | postive |
| TRPC6 | TRAF3IP2-AS1 | 0.422635486 | 4.19E-09 | postive |
| TRPC7 | TRAF3IP2-AS1 | 0.507609288 | 4.77E-13 | postive |
| TRPM4 | TRAF3IP2-AS1 | -0.406539339 | 1.79E-08 | negative |
| TRPM5 | TRAF3IP2-AS1 | 0.442754598 | 6.08E-10 | postive |
| TRPM6 | TRAF3IP2-AS1 | 0.430704002 | 1.96E-09 | postive |
| MCOLN1 | TRAF3IP2-AS1 | 0.573043573 | 6.35E-17 | postive |
| MCOLN3 | TRAF3IP2-AS1 | 0.720139067 | 9.54E-30 | postive |
| PKD1 | TRAF3IP2-AS1 | 0.514294213 | 2.09E-13 | postive |
| PKD2 | TRAF3IP2-AS1 | 0.409182091 | 1.42E-08 | postive |
| TRPA1 | AL596442.2 | 0.421427109 | 4.68E-09 | postive |
| TRPM2 | AL596442.2 | 0.438941178 | 8.85E-10 | postive |
| TRPC1 | AC005332.6 | 0.610288957 | 1.52E-19 | postive |
| TRPC6 | AC005332.6 | 0.432304911 | 1.68E-09 | postive |
| TRPM7 | AC005332.6 | 0.590779809 | 3.95E-18 | postive |
| PKD2 | AC005332.6 | 0.669696875 | 1.61E-24 | postive |
| TRPM5 | AC009019.1 | 0.428027087 | 2.53E-09 | postive |
| MCOLN3 | AC009019.1 | 0.439498894 | 8.38E-10 | postive |
| PKD1 | AC009019.1 | 0.443827468 | 5.47E-10 | postive |
| TRPV1 | AC009019.1 | 0.412106762 | 1.09E-08 | postive |
| TRPC3 | DIRC3 | 0.448692255 | 3.36E-10 | postive |
| TRPC7 | AC092171.3 | 0.462987314 | 7.66E-11 | postive |
| TRPM5 | AC092171.3 | 0.512611859 | 2.58E-13 | postive |
| MCOLN3 | AC092171.3 | 0.500735166 | 1.10E-12 | postive |
| PKD1 | AC092171.3 | 0.516190334 | 1.65E-13 | postive |
| TRPC1 | AC022098.1 | 0.421950283 | 4.46E-09 | postive |
| MCOLN1 | AC022098.1 | 0.447325201 | 3.85E-10 | postive |
| PKD1 | AC022098.1 | 0.438580595 | 9.17E-10 | postive |
| TRPV1 | AC022098.1 | 0.464189379 | 6.74E-11 | postive |
| PKD1 | AL021368.1 | 0.454302938 | 1.90E-10 | postive |
| TRPC4 | AC005062.1 | 0.509239011 | 3.91E-13 | postive |
| TRPC7 | AC005062.1 | 0.68402265 | 6.75E-26 | postive |
| TRPM5 | AC098828.1 | 0.645696769 | 2.23E-22 | postive |
| MCOLN3 | AC098828.1 | 0.489813311 | 3.95E-12 | postive |
| TRPV1 | U73169.1 | 0.461041726 | 9.41E-11 | postive |
| TRPC1 | AL122010.1 | 0.445563007 | 4.60E-10 | postive |
| TRPC4 | AL122010.1 | 0.422900619 | 4.08E-09 | postive |
| TRPM5 | AL122010.1 | 0.541854424 | 5.69E-15 | postive |
| MCOLN3 | AL122010.1 | 0.618966581 | 3.32E-20 | postive |
| PKD1 | AL122010.1 | 0.567414058 | 1.48E-16 | postive |
| TRPC7 | AC005498.2 | 0.553176863 | 1.17E-15 | postive |
| TRPM5 | AC005498.2 | 0.470759302 | 3.33E-11 | postive |
| MCOLN1 | AC005498.2 | 0.599624049 | 9.27E-19 | postive |
| MCOLN3 | AC005498.2 | 0.641863299 | 4.72E-22 | postive |
| PKD1 | AC005498.2 | 0.459587002 | 1.10E-10 | postive |
| TRPC4 | AC021242.3 | 0.469544234 | 3.80E-11 | postive |
| TRPC7 | AC021242.3 | 0.664131562 | 5.26E-24 | postive |
| TRPM3 | AC021242.3 | 0.508667217 | 4.19E-13 | postive |
| MCOLN1 | AC021242.3 | 0.513747372 | 2.24E-13 | postive |
| MCOLN3 | AC021242.3 | 0.741056701 | 2.89E-32 | postive |
| PKD1 | AC021242.3 | 0.410887047 | 1.22E-08 | postive |
| TRPV1 | GUSBP11 | 0.538089254 | 9.49E-15 | postive |
| MCOLN2 | AC008649.1 | 0.418818332 | 5.95E-09 | postive |
| TRPM5 | AC009159.3 | 0.634037765 | 2.10E-21 | postive |
| MCOLN3 | AC009159.3 | 0.657444998 | 2.11E-23 | postive |
| PKD1 | AC009159.3 | 0.413039089 | 1.01E-08 | postive |
| TRPC2 | LINC02611 | 0.560622537 | 4.02E-16 | postive |
| TRPC6 | LINC02611 | 0.401822314 | 2.71E-08 | postive |
| TRPM2 | LINC02611 | 0.591924305 | 3.28E-18 | postive |
| MCOLN2 | LINC02611 | 0.590793531 | 3.94E-18 | postive |
| PKD2 | LINC02611 | 0.434410464 | 1.37E-09 | postive |
| PKD2L1 | LINC02611 | 0.468235846 | 4.38E-11 | postive |
| TRPV2 | LINC02611 | 0.511829356 | 2.84E-13 | postive |
| TRPC3 | AC087672.2 | 0.440830616 | 7.35E-10 | postive |
| PKD2 | AC087672.2 | 0.419136343 | 5.78E-09 | postive |
| TRPC7 | AC090241.3 | 0.493997911 | 2.43E-12 | postive |
| PKD1 | AC090241.3 | 0.554502791 | 9.72E-16 | postive |
| MCOLN2 | LINC02391 | 0.665583079 | 3.87E-24 | postive |
| TRPC2 | AC002091.1 | 0.411285429 | 1.18E-08 | postive |
| TRPC6 | AC002091.1 | 0.422940233 | 4.07E-09 | postive |
| TRPM2 | AC002091.1 | 0.635474866 | 1.60E-21 | postive |
| MCOLN2 | AC002091.1 | 0.580711119 | 1.95E-17 | postive |
| PKD2 | AC002091.1 | 0.458737864 | 1.20E-10 | postive |
| PKD2L1 | AC002091.1 | 0.50350661 | 7.86E-13 | postive |
| TRPC1 | LRRC8C-DT | 0.406800915 | 1.75E-08 | postive |
| TRPC6 | LRRC8C-DT | 0.463939207 | 6.93E-11 | postive |
| MCOLN2 | LRRC8C-DT | 0.588584086 | 5.62E-18 | postive |
| TRPA1 | AC009065.2 | 0.48379524 | 7.86E-12 | postive |
| TRPC1 | AC009065.2 | -0.412109879 | 1.09E-08 | negative |
| TRPM4 | AC009065.2 | 0.568559707 | 1.25E-16 | postive |
| TRPC1 | AC006116.5 | 0.40700473 | 1.72E-08 | postive |
| TRPC1 | AL390208.1 | 0.403130467 | 2.42E-08 | postive |
| MCOLN1 | AL390208.1 | 0.403324096 | 2.38E-08 | postive |
| MCOLN2 | AL390208.1 | 0.465138786 | 6.10E-11 | postive |
| PKD1 | AL390208.1 | 0.566464109 | 1.70E-16 | postive |
| TRPV1 | AL390208.1 | 0.408878809 | 1.46E-08 | postive |
| TRPV6 | AL355300.1 | 0.410276522 | 1.29E-08 | postive |
| TRPC1 | AC009812.1 | 0.530107745 | 2.75E-14 | postive |
| TRPC7 | AC009812.1 | 0.621988407 | 1.93E-20 | postive |
| TRPM3 | AC009812.1 | 0.427448454 | 2.67E-09 | postive |
| TRPM5 | AC009812.1 | 0.540932142 | 6.45E-15 | postive |
| MCOLN1 | AC009812.1 | 0.526484422 | 4.42E-14 | postive |
| MCOLN3 | AC009812.1 | 0.732401621 | 3.40E-31 | postive |
| PKD1 | AC009812.1 | 0.536794749 | 1.13E-14 | postive |
| MCOLN2 | SATB1-AS1 | 0.627929047 | 6.55E-21 | postive |
| MCOLN2 | MIR155HG | 0.864272852 | 2.09E-54 | postive |
